# Supplementary material for: Gaps in protection to Anopheles exposure in high malaria endemic regencies of Papua Province, Indonesia
Source: PLoS One. 2025 Apr 11;20(4):e0311076. doi: 10.1371/journal.pone.0311076 (PMC11990486; doi:10.1371/journal.pone.0311076)
Supplement: S1 File — (PDF) [file pone.0311076.s001.pdf]

Supplementary File 1

List of Questionnaires

Part A. Questionnaire for Human behavior observation (HBO)

Each question was answered hourly during HLC activity in each sentinel house and data recorded based on observation

1. How many are people under bed nets indoor?
2. How many people are NOT under bed nets indoors?
3. How many people are awake outdoors?
4. How many people are asleep outdoors?

Part B. Questionnaire for Household survey

1) Household general information
   a. Address of the household (province, regency, district, village and hamlet)
   b. GPS coordinates of the household
   c. Total household members
   d. Interviewee general information (name, age, relationship to household head and last education)
2) Building structure information
   a. Wall material (multiple choice)
   b. Roof material (multiple choice)
   c. Floor material (multiple choice)
   d. Total bedroom
   e. Distance floor to the land (multiple choice)

f. Door number (screened or not)
g. Window number (screened or not)
h. Eaves (screened or not)
i. Fire burning inside the house (Yes/No)
j. Fire burning outside the house (Yes/No)

3) Socio economic status

a. Household head main job (multiple choice)
b. Electrical source (multiple choice)
c. Asset of household (multiple choice)
d. Vehicles (multiple choice)
e. Livestock (multiple choice)
f. Cooking fuel (multiple choice)
g. Water source (multiple choice)
h. Distance of water source (multiple choice)
i. Toilet facility (multiple choice)
j. Bathroom facility (multiple choice)

4) Protection against mosquito bites

a. Indoor residual spraying (IRS) (last IRS information)
b. Used of insect repellent (multiple choice)
c. Total number of bed nets
d. Who are sleeping under bed net (multiple choice)
e. People used bed net last night (multiple choice)
f. Reasons not using bed net last night (multiple choice)

5) Bed net condition

a. The origin of the bednet is obtained (multiple choice)

b. Age of the bed net

c. Insecticide net (Yes/No)

d. Bed net condition (multiple choice)

e. The bed net hung last night (Yes/No)

6) Human behavior against mosquito bites

a. Dinner time (multiple choice)

b. Dinner place (multiple choice)

c. Resting after dinner (multiple choice)

d. Sleeping time (multiple choice)

e. Waking up time (multiple choice)

f. Sleeping place (multiple choice)

g. Sleeping under bed net (multiple choice)

h. Going outdoor at night (where to go, how long, use repellent outside)

7) Malaria information

a. Person getting malaria in the household (multiple choice)

b. Last malaria case in the household (multiple choice)

c. How to know malaria (multiple choice)

d. Death caused by malaria in recent 2 years (Yes/No)
