# Supplementary material for: Gaps in protection to Anopheles exposure in high malaria endemic regencies of Papua Province, Indonesia
Source: PLoS One. 2025 Apr 11;20(4):e0311076. doi: 10.1371/journal.pone.0311076 (PMC11990486; doi:10.1371/journal.pone.0311076)
Supplement: S2 File — (PDF) [file pone.0311076.s002.pdf]

# Supplementary File 2

## Human behavior observations and adjusted-human biting rates in 14 villages

1. Skofro Village, Keerom Regency (Inland, HBR = 58.0 bpn)

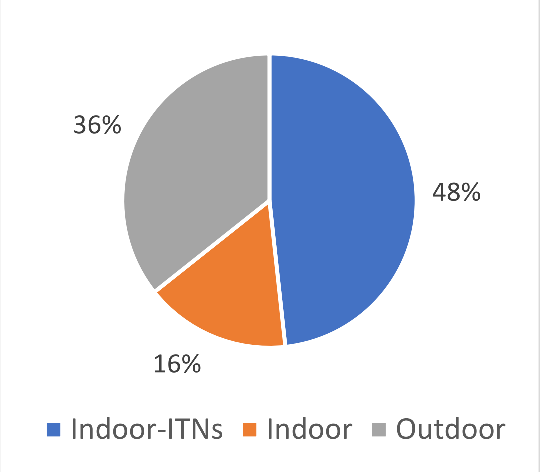


Figure S2-1a. Total human behavior proportion from Skofro Village

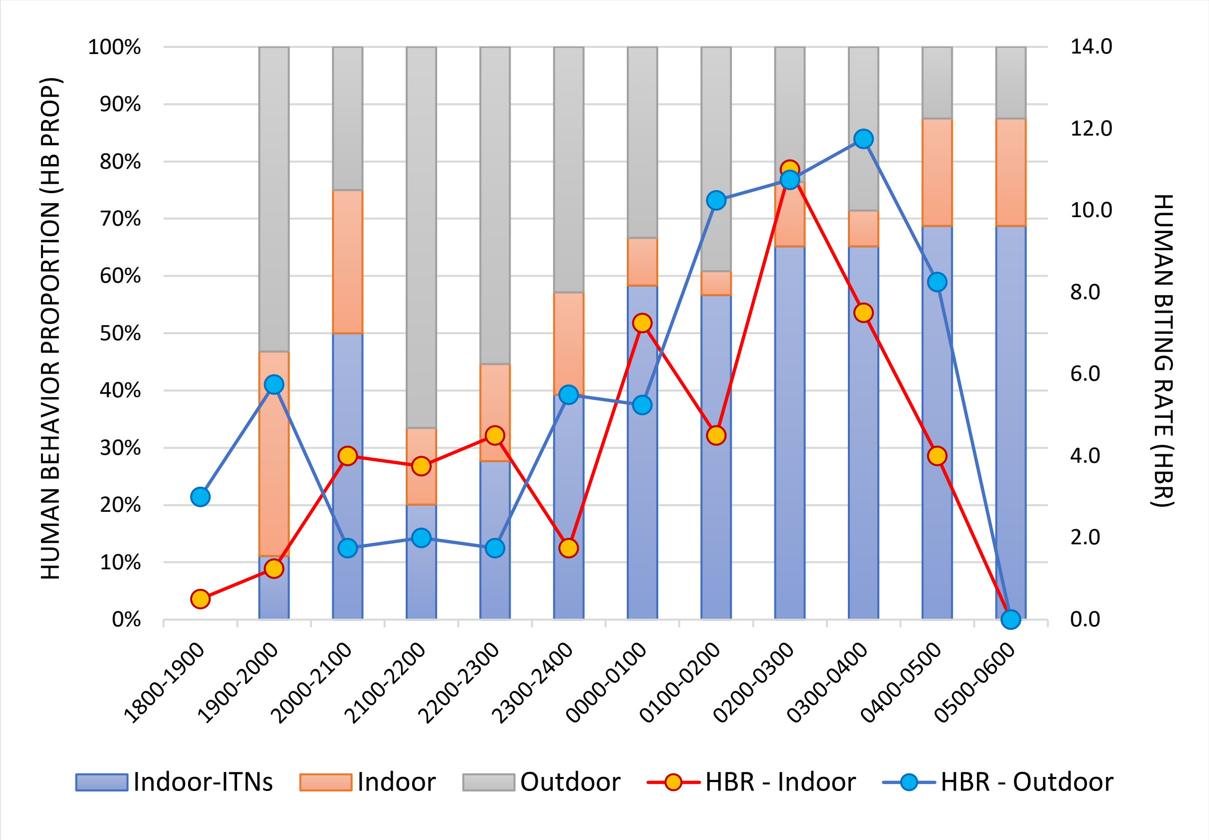


Figure S2-1b. Proportion of HBO vs indoor and outdoor HBR (bph) from Skofro Village

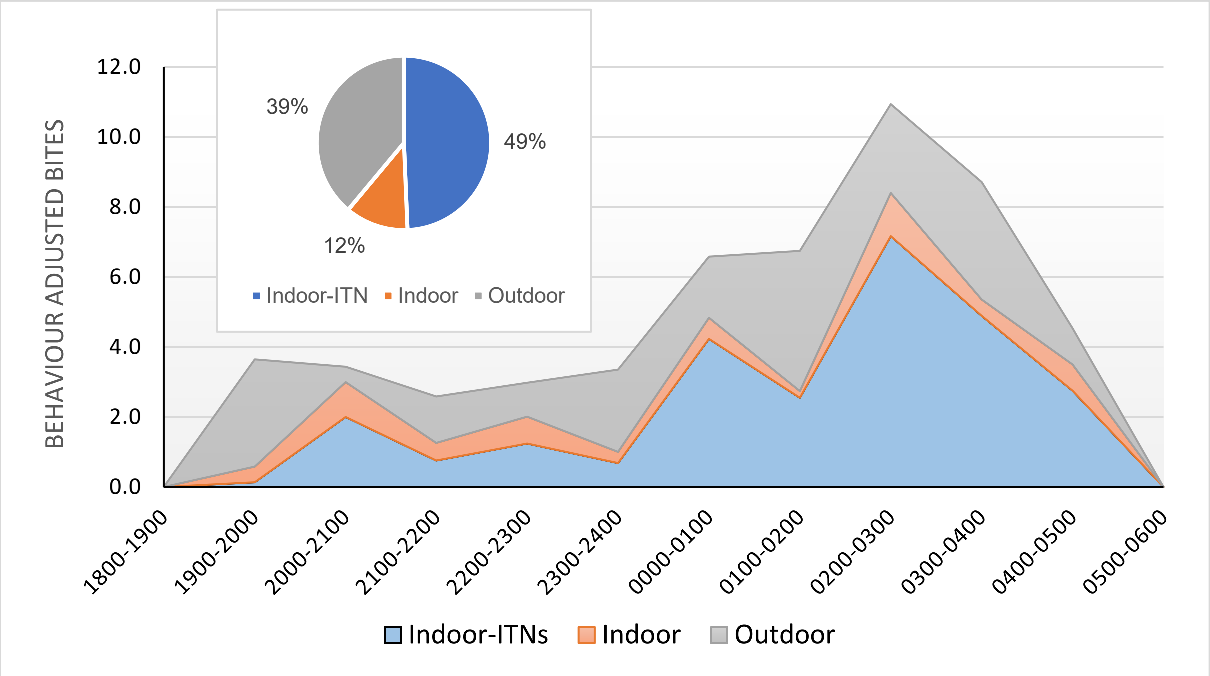


Figure S2-1c. Human Behavior Adjusted from Skofro Village

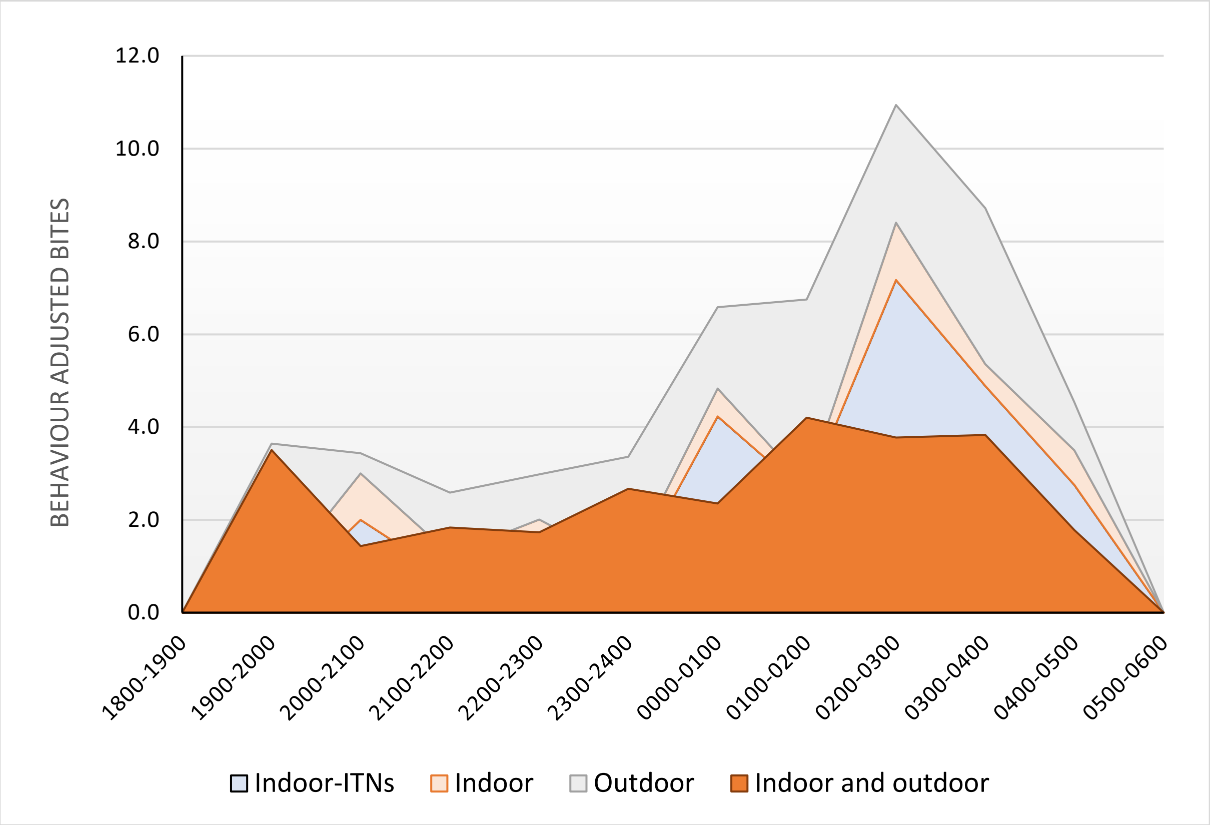


Figure S2-1d. Behavior-adjusted exposure rate for an unprotected individual from Skofro Village

2. Pikere Village, Keerom Regency (Inland, HBR = 39.6 bpn)

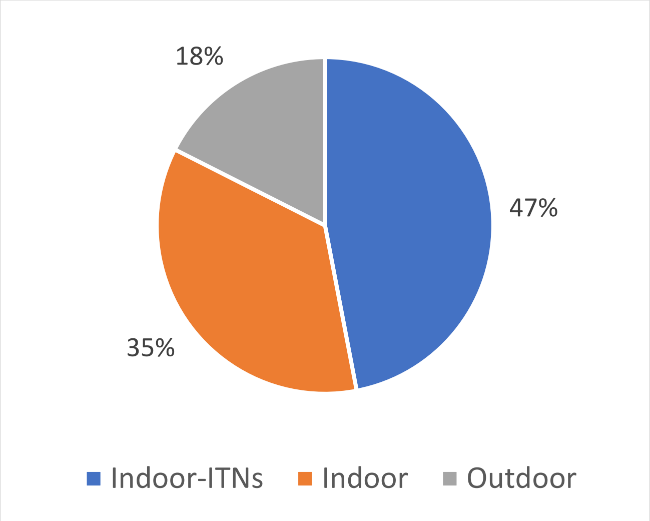


Figure S2-2a. Total human behavior proportion from Pikere Village

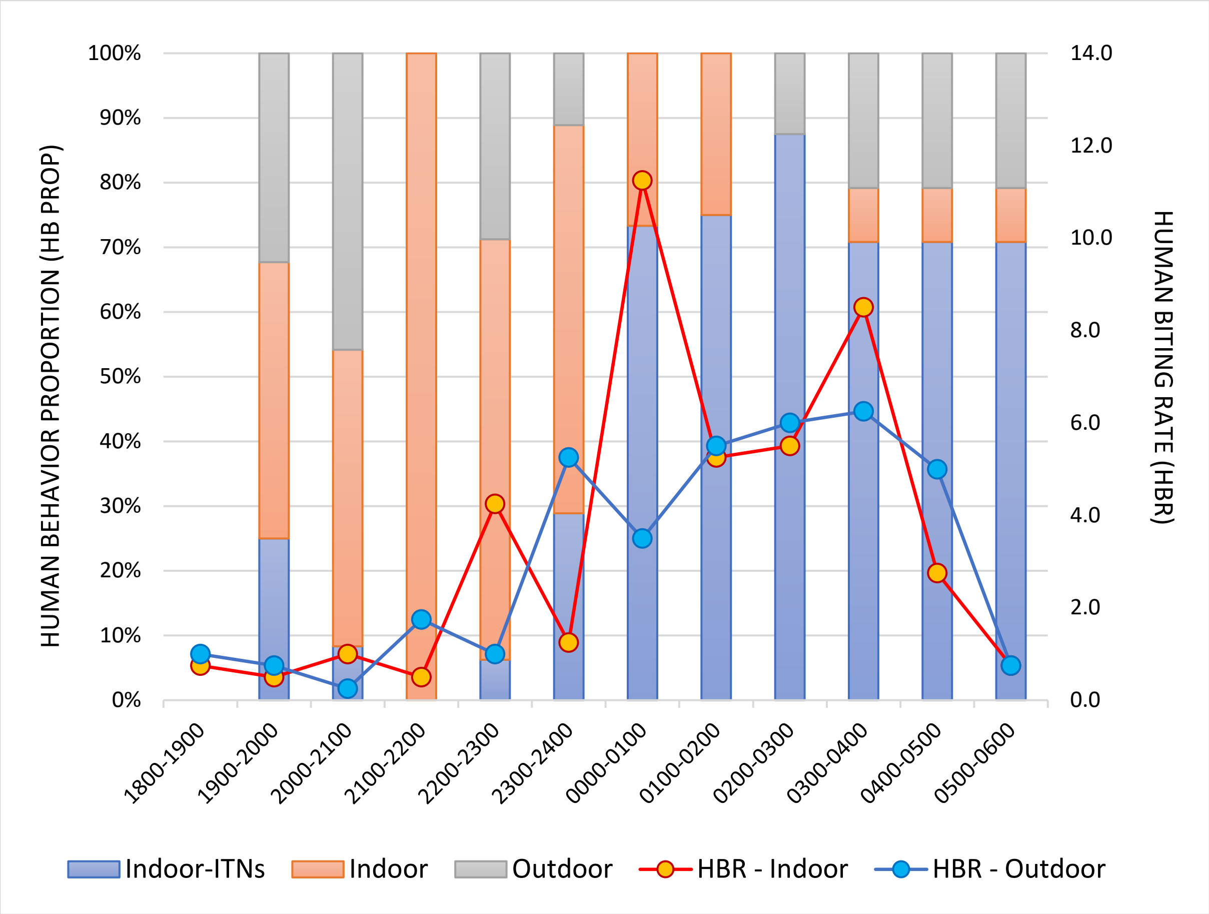


Figure S2-2b. Proportion of HBO vs indoor and outdoor HBR (bph) from Pikere Village

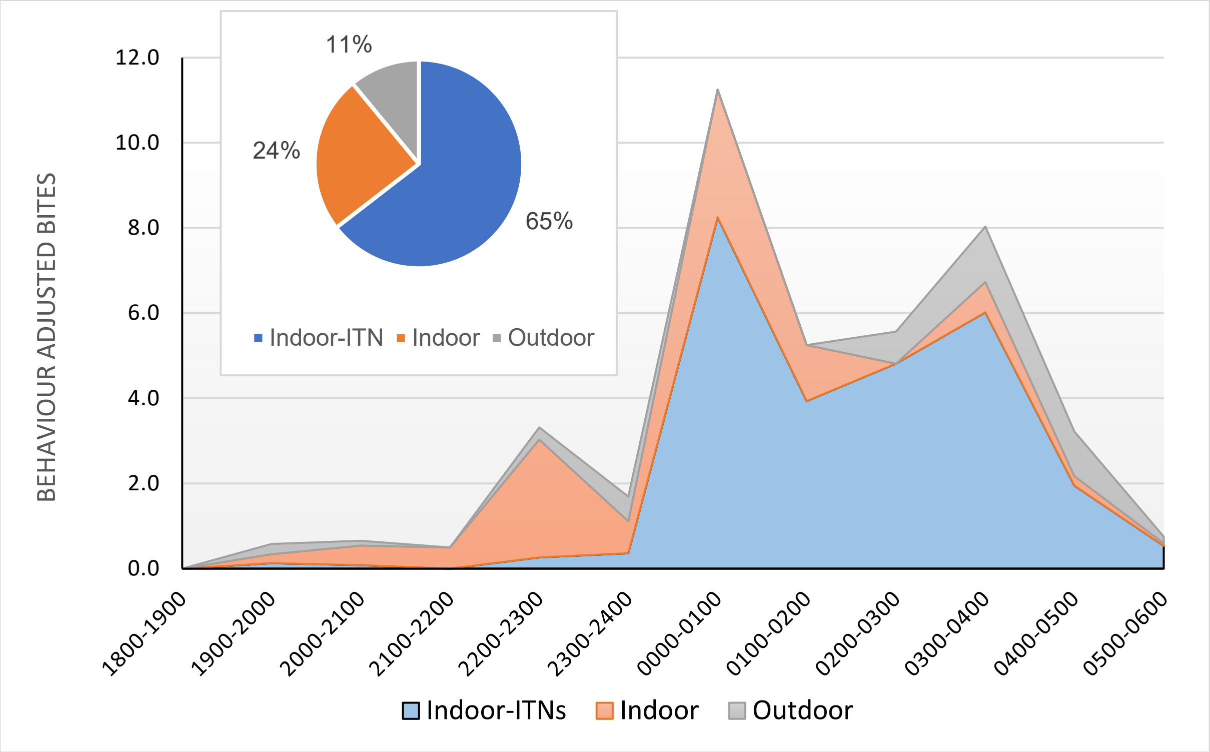


Figure S2-2c. Human Behavior Adjusted from Pikere Village

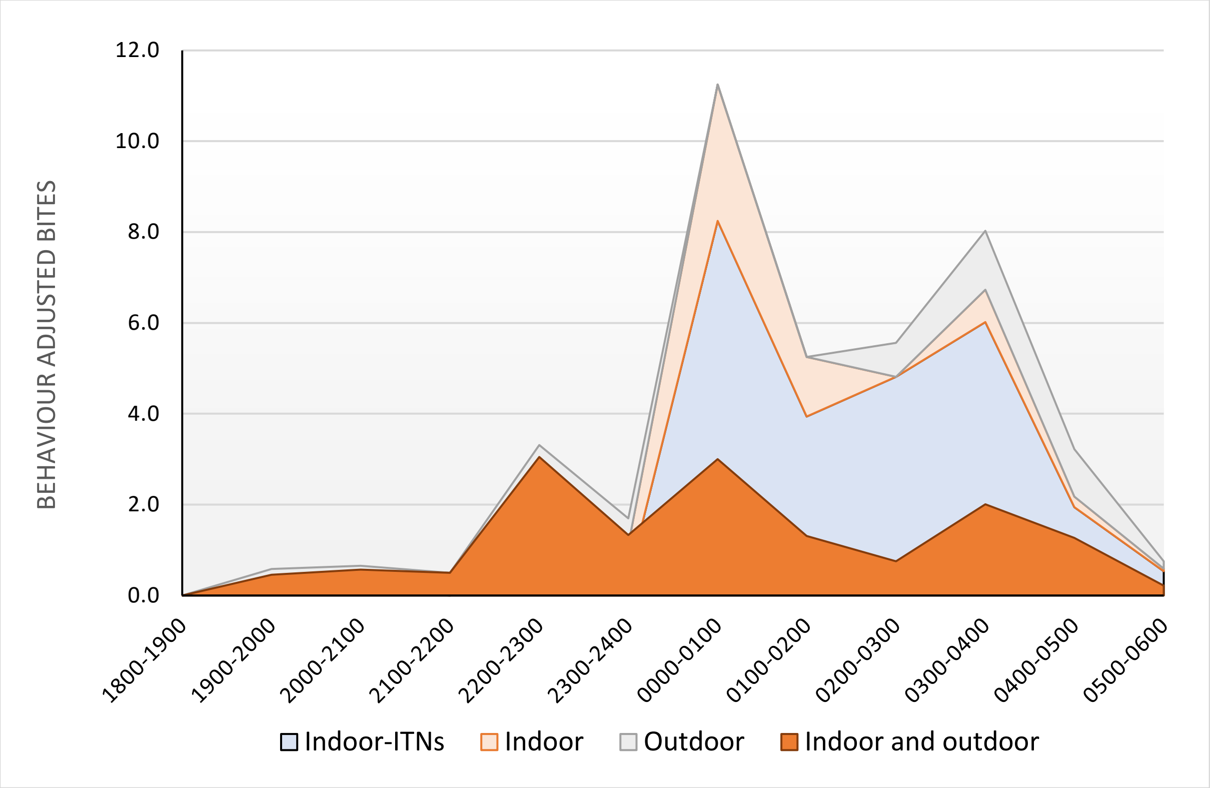


Figure S2-2d. Behavior-adjusted exposure rate for an unprotected individual from Pikere Village

3. Hanggey Among Village, Jayapura Regency (Inland, HBR = 66.9 bpn)

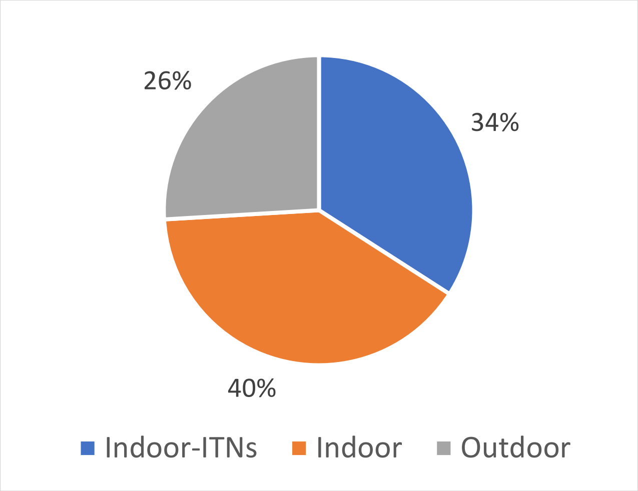


Figure S2-3a. Total human behavior proportion from Hanggey Among Village

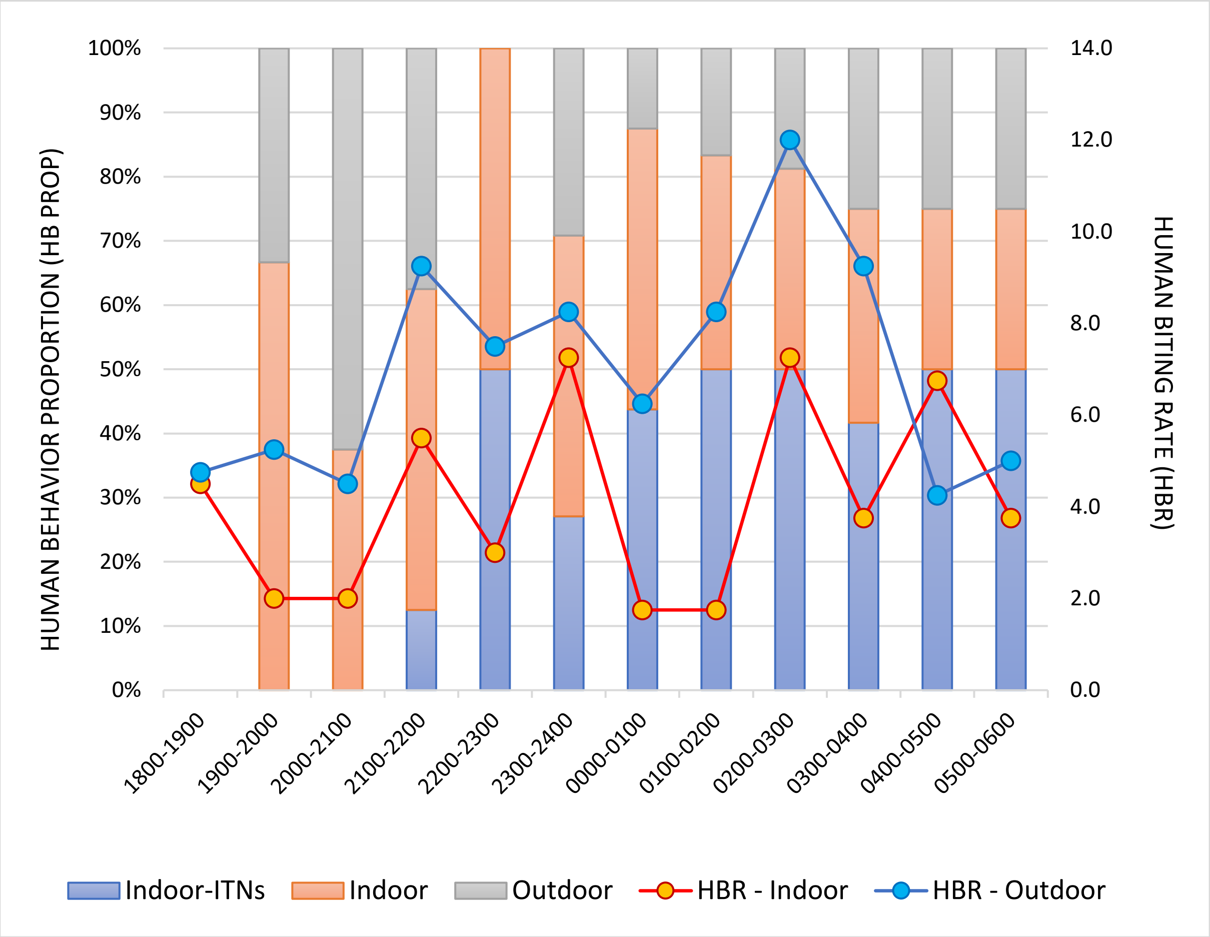


Figure S2-3b. Proportion of HBO vs indoor and outdoor HBR (bph) from Hanggey Among Village

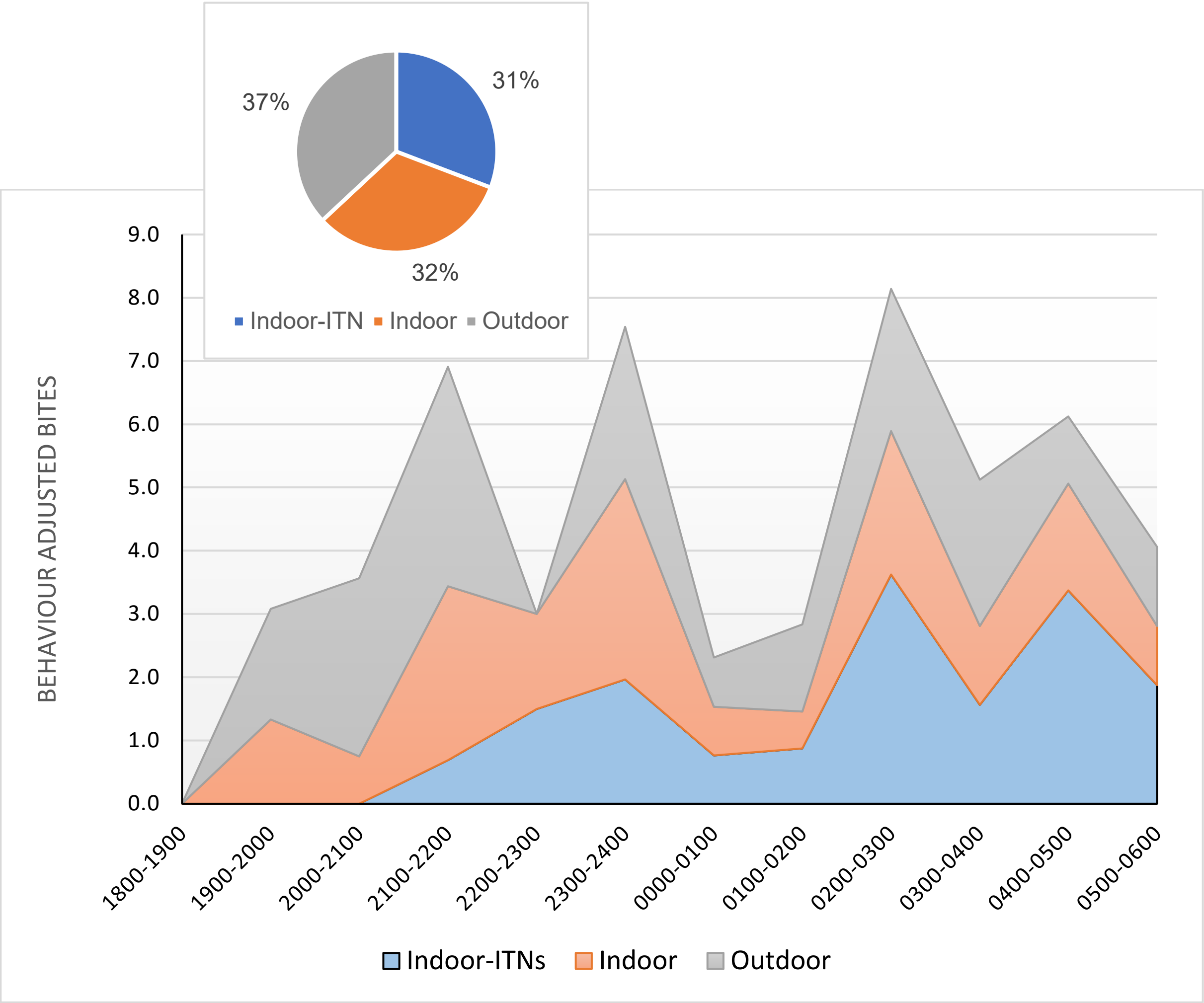


Figure S2-3c. Human Behavior Adjusted from Hanggey Among Village

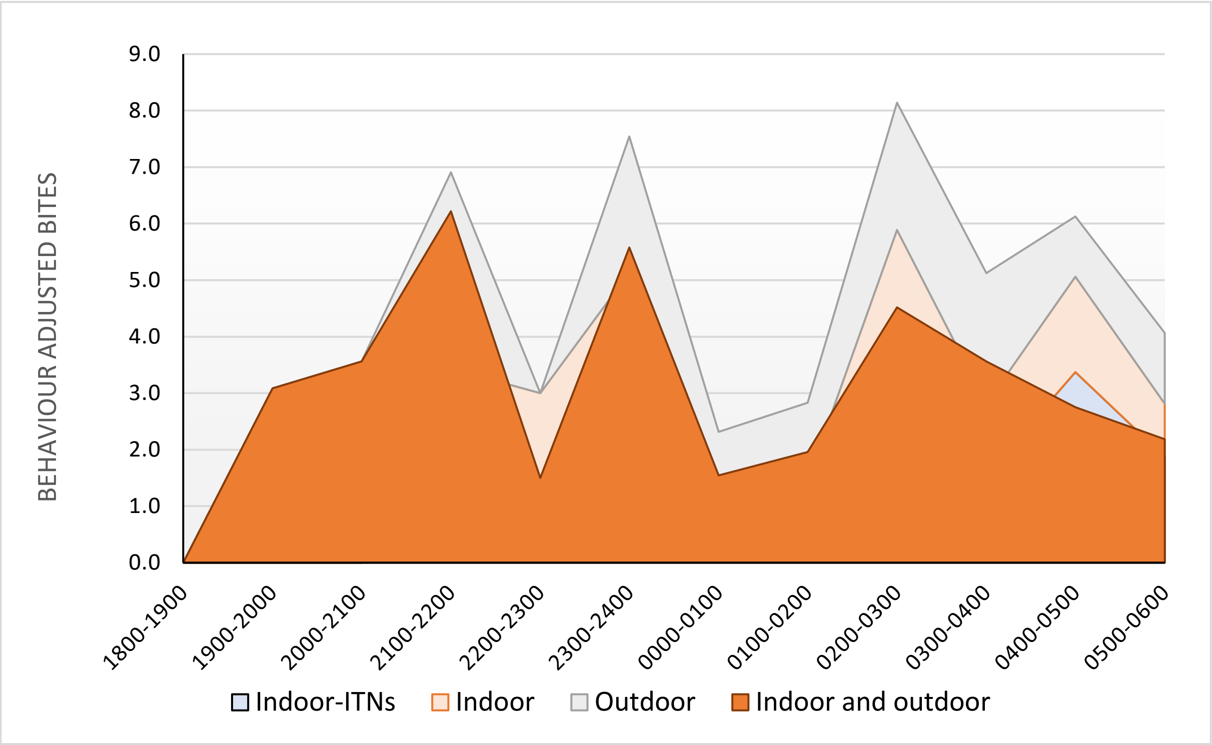


Figure S2-3d. Behavior-adjusted exposure rate for an unprotected individual from Hanggey Among Village

4. Sunum/Yamna Village, Sarmi Regency (Coastal, HBR = 30.0 bpn)

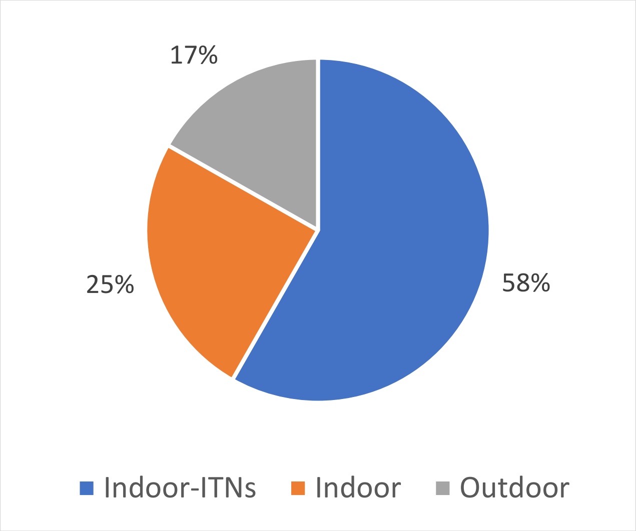


Figure S2-4a. Total human behavior proportion from Sunum/Yamna Village

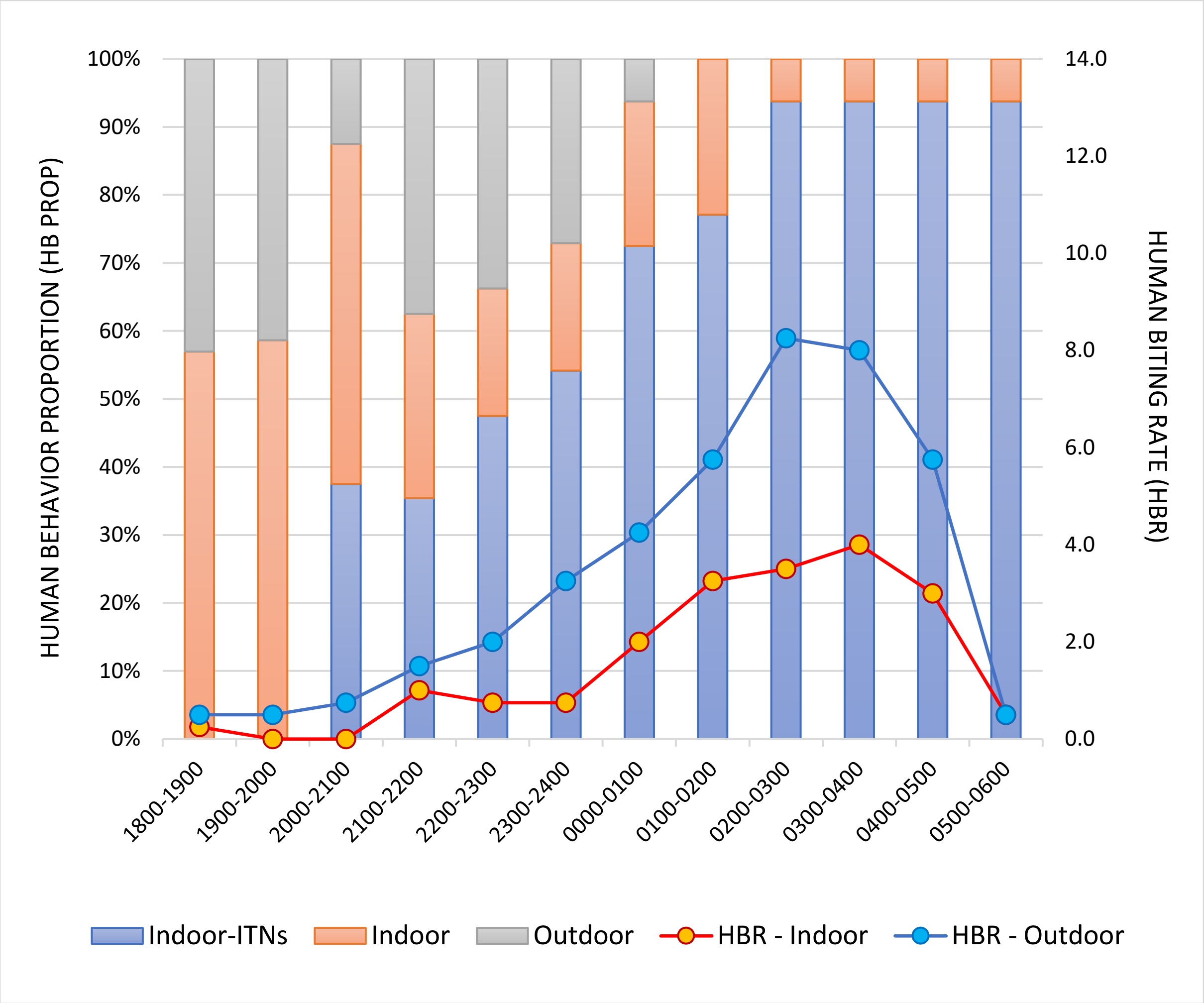


Figure S2-4b. Proportion of HBO vs indoor and outdoor HBR (bph) from Sunum/Yamna Village

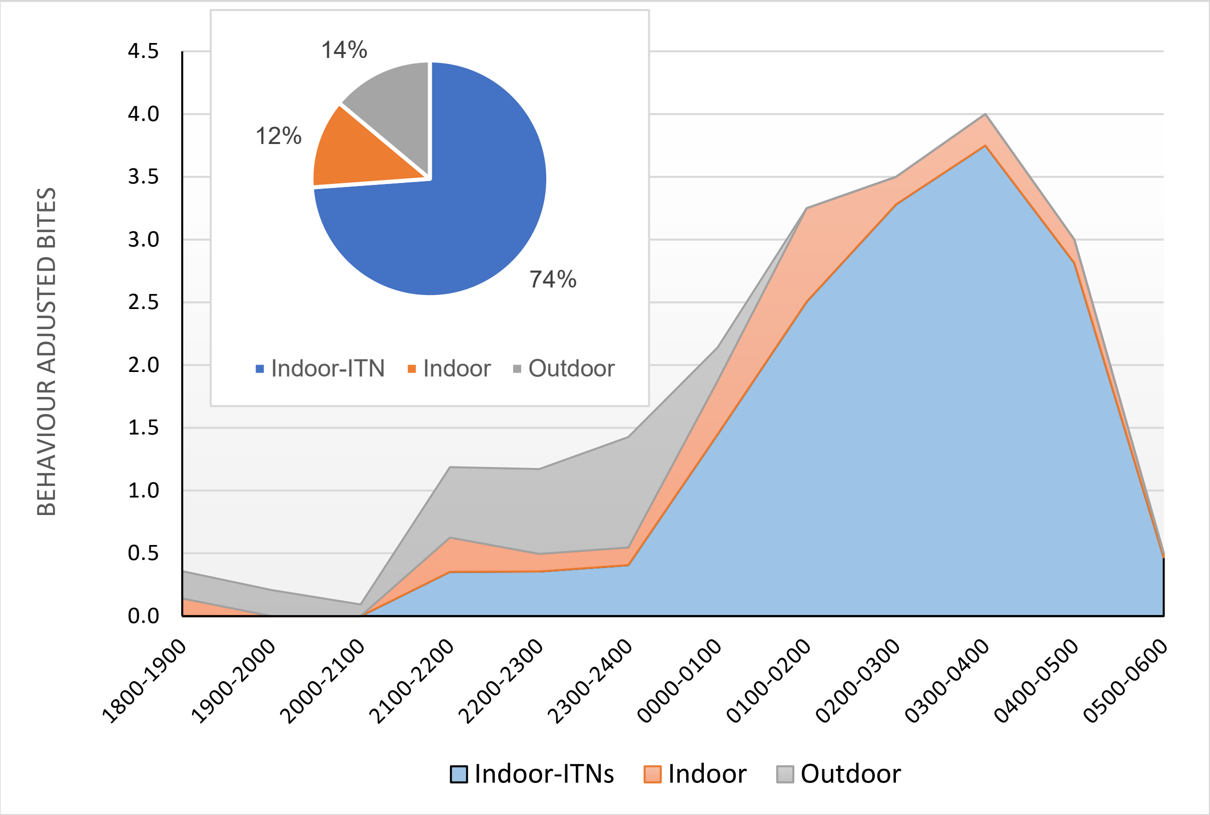


Figure S2-4c. Human Behavior Adjusted from Sunum/Yamna Village

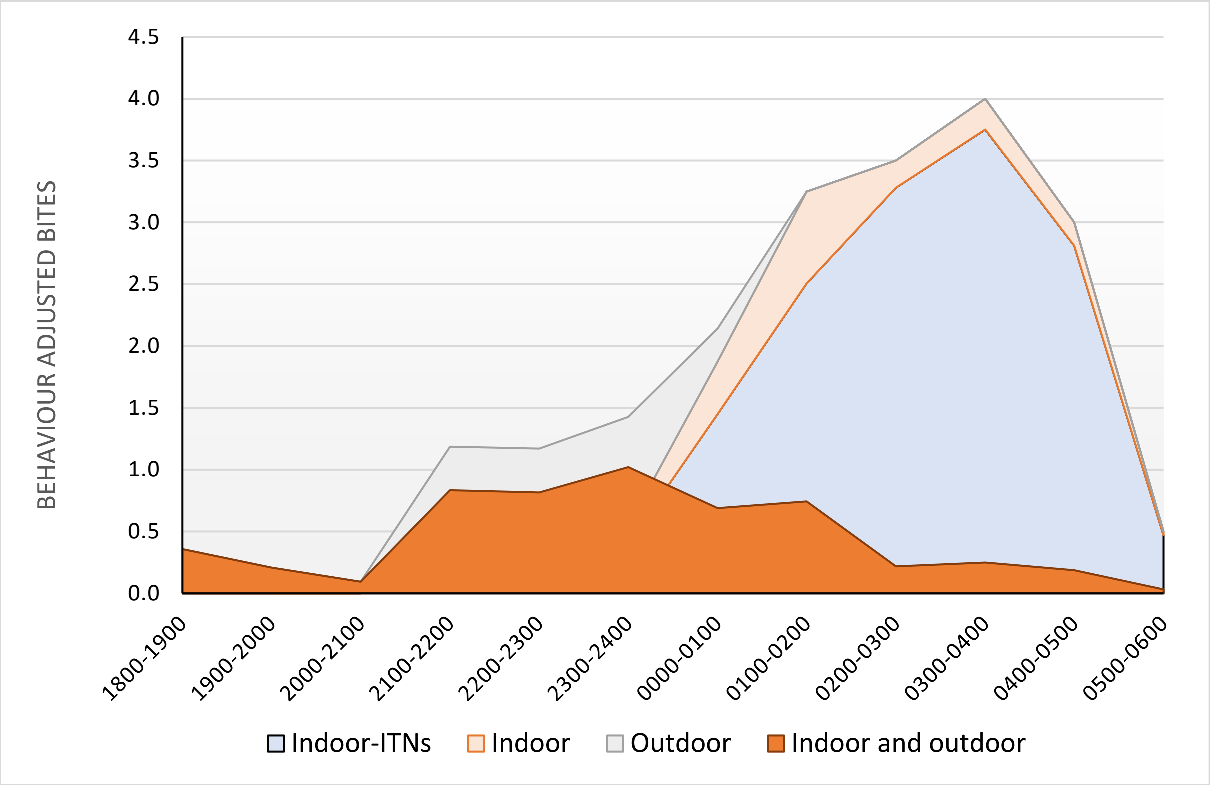


Figure S2-4d. Behavior-adjusted exposure rate for an unprotected individual from Sunum/Yamna Village

5. Samanente Village, Sarmi Regency (Inland, HBR = 11.5 bpn)

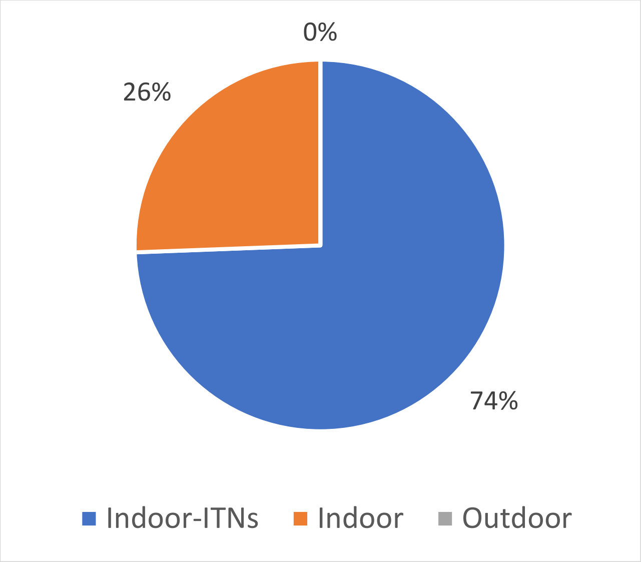


Figure S2-5a. Total human behavior proportion from Samanente Village

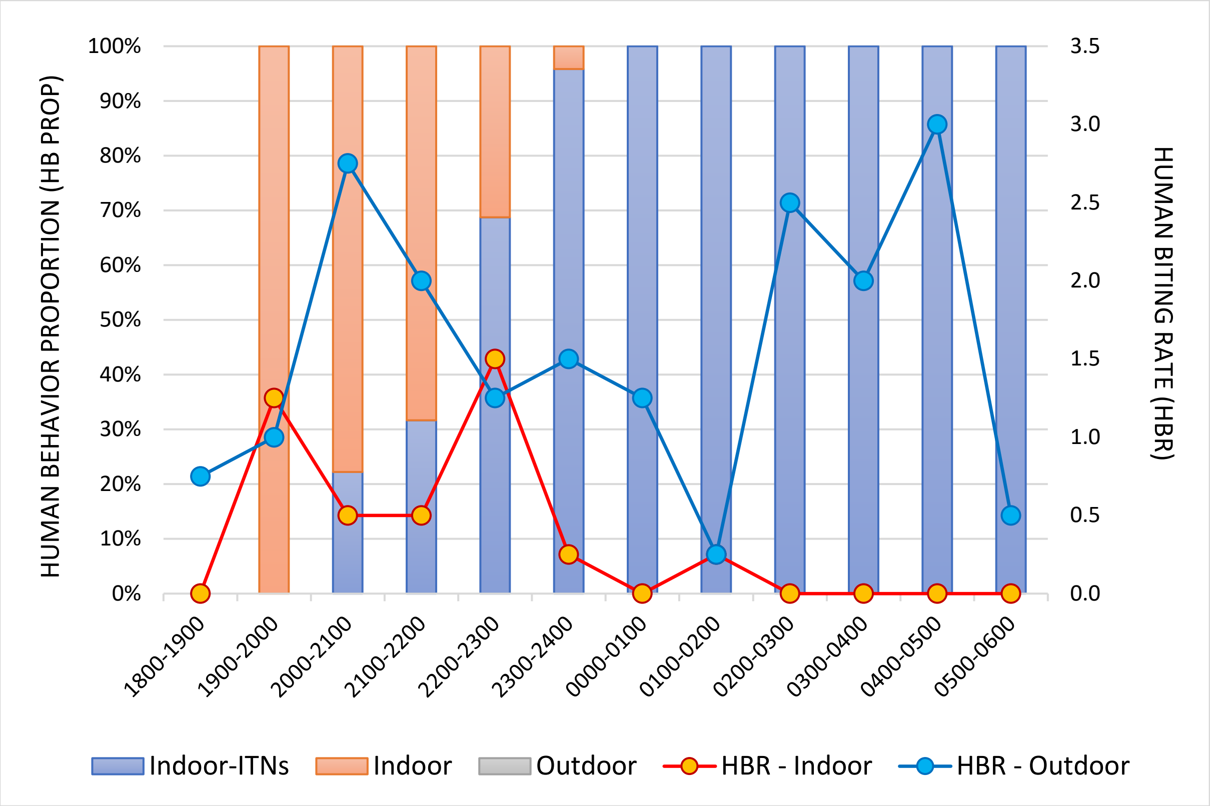


Figure S2-5b. Proportion of HBO vs indoor and outdoor HBR (bph) from Samanente Village

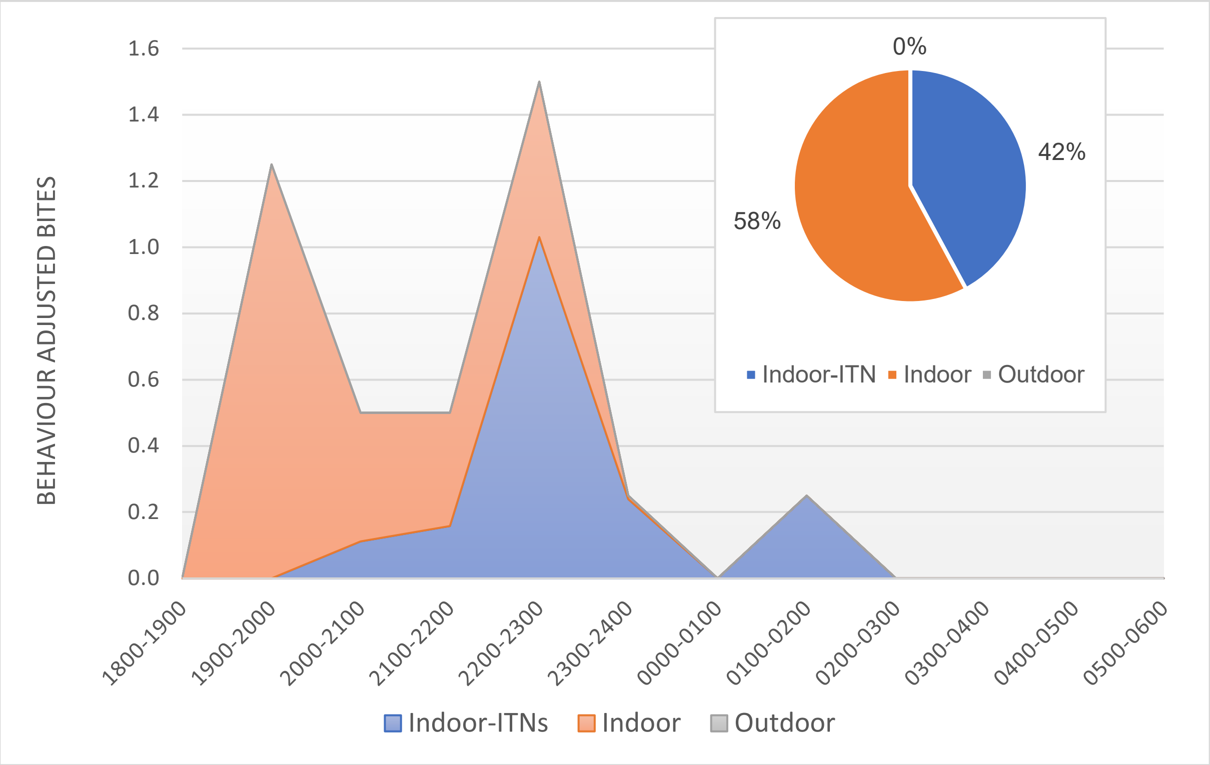


Figure S2-5c. Human Behavior Adjusted from Samanente Village

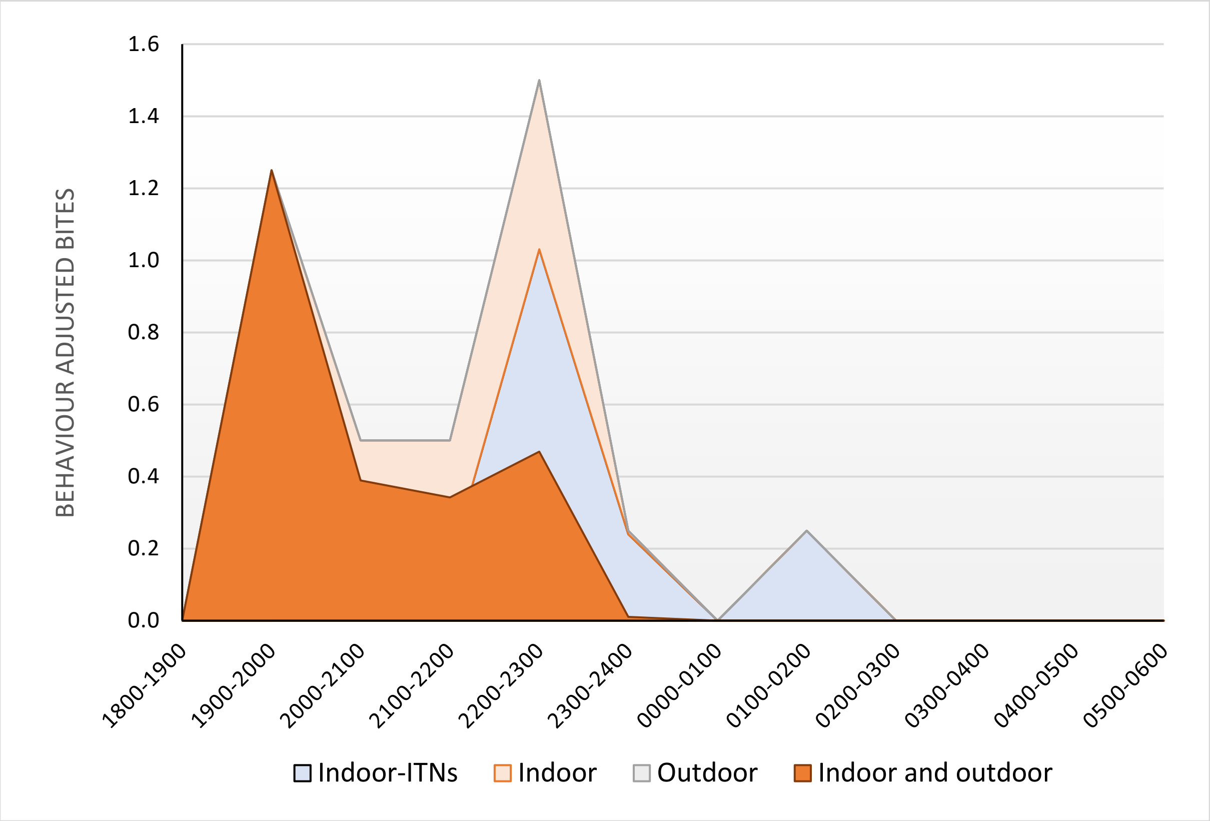


Figure S2-5d. Behavior-adjusted exposure rate for an unprotected individual from Samanente Village

6. Konderjan Village, Sarmi Regency (Inland, HBR = 9.5 bpn)

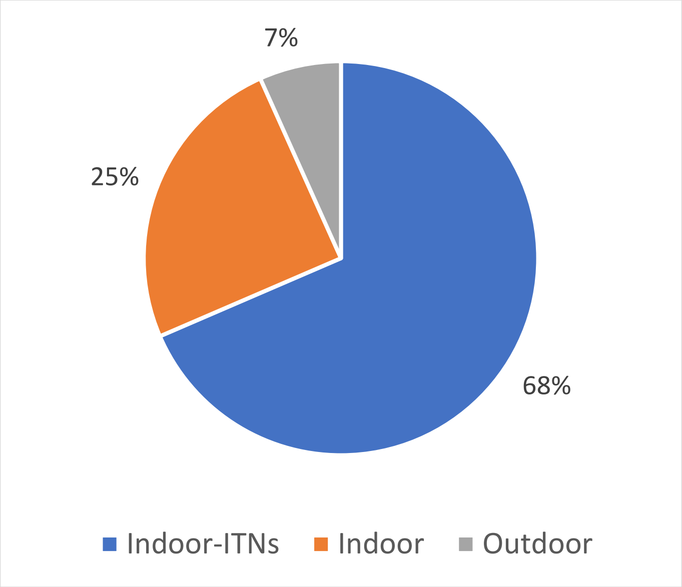


Figure S2-6a. Total human behavior proportion from Konderjan Village

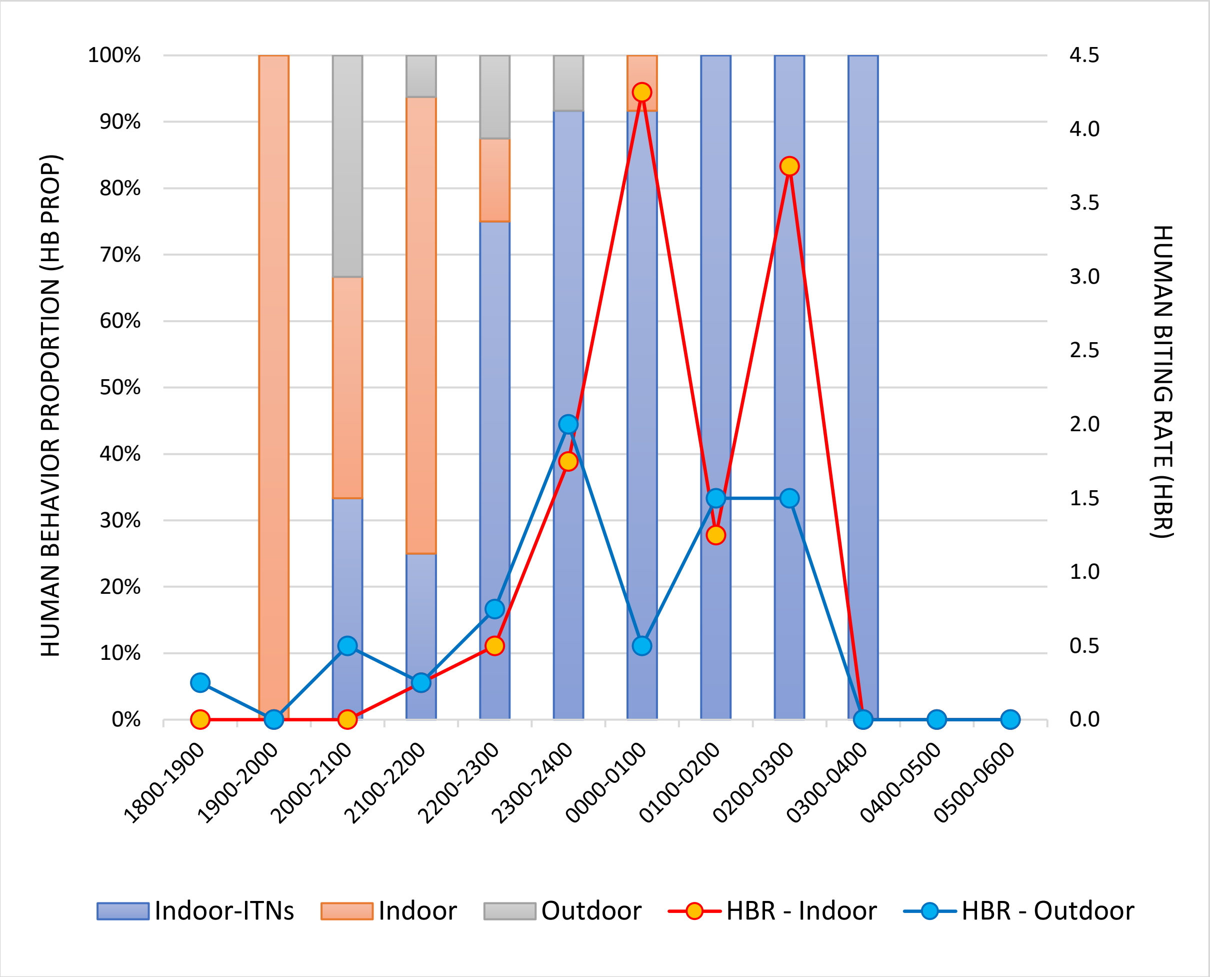


Figure S2-6b. Proportion of HBO vs indoor and outdoor HBR (bph) from Konderjan Village

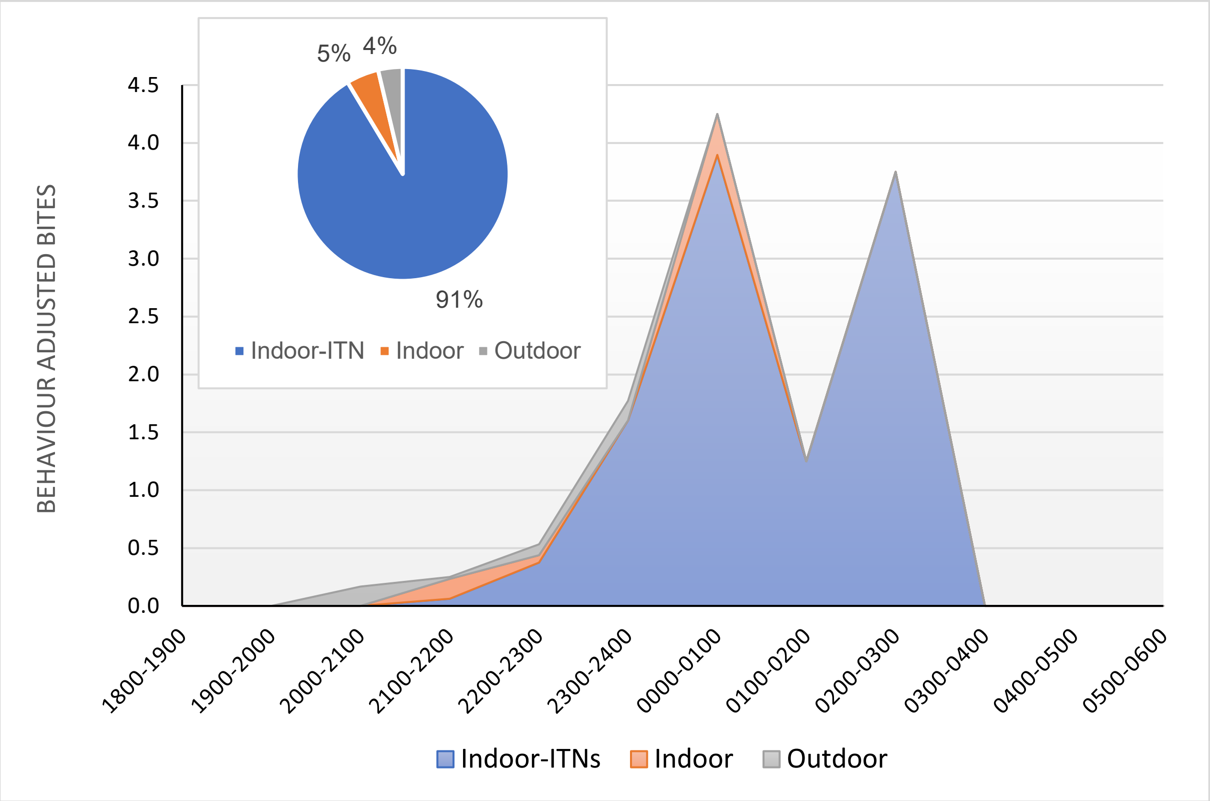


Figure S2-6c. Human Behavior Adjusted from Konderjan Village

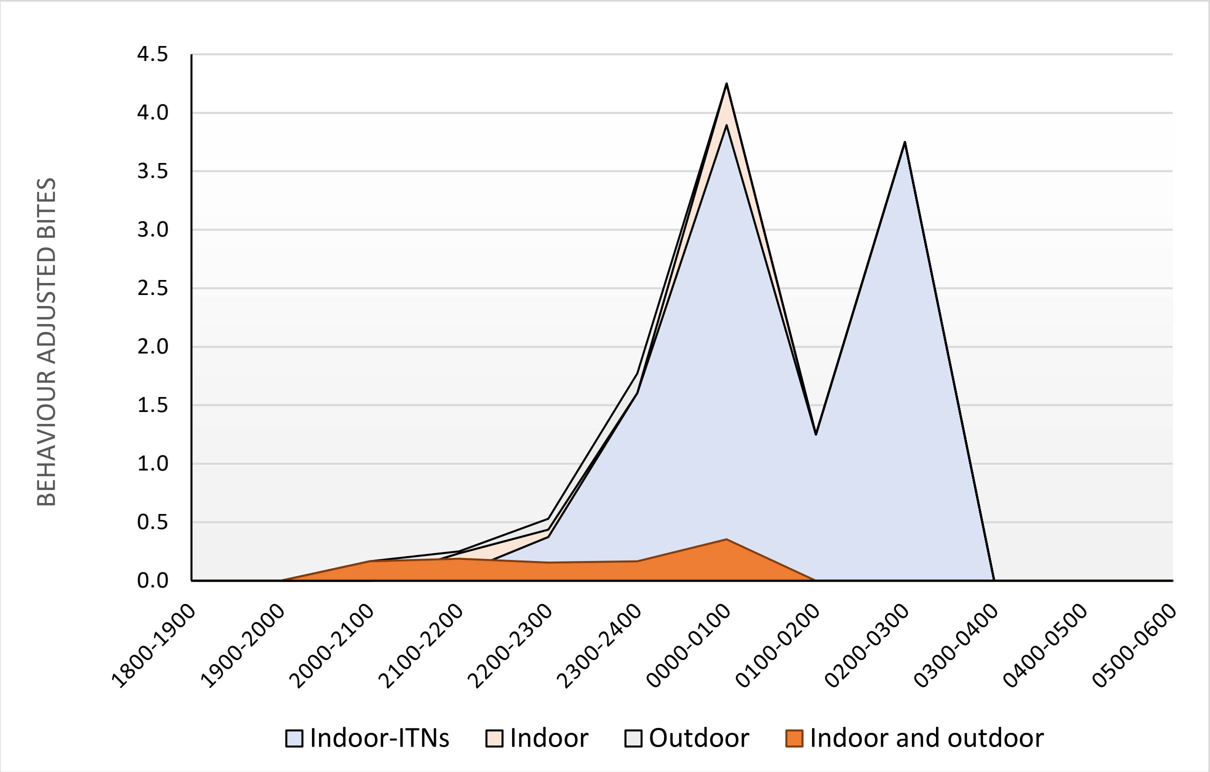


Figure S2-6d. Behavior-adjusted exposure rate for an unprotected individual from Konderjan Village

7. Arbais Village, Sarmi Regency (Coastal, HBR = 21.0 bpn)

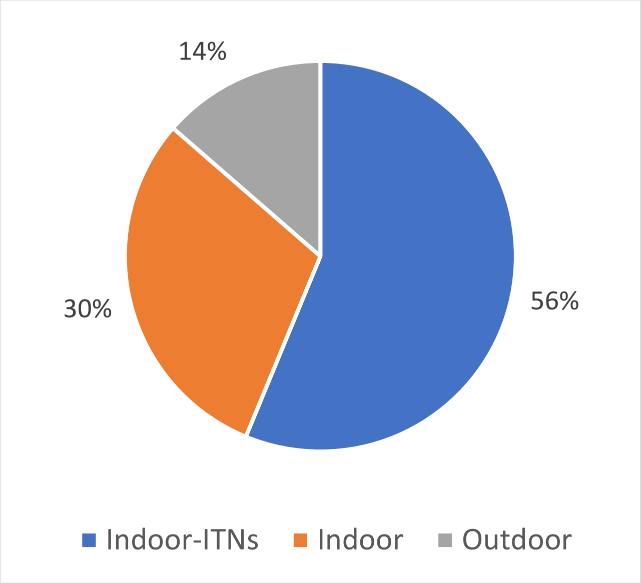


Figure S2-7a. Total human behavior proportion from Arbais Village

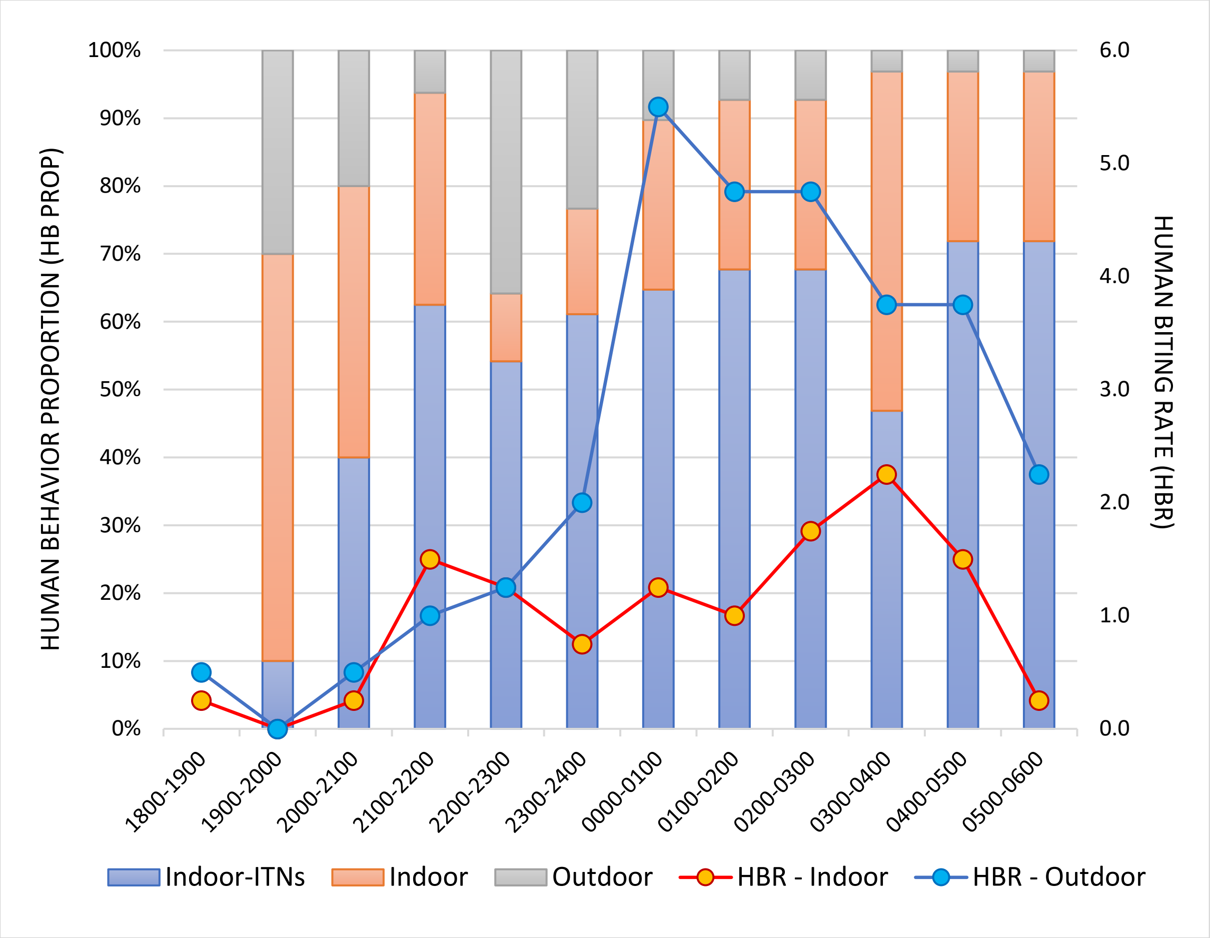


Figure S2-7b. Proportion of HBO vs indoor and outdoor HBR (bph) from Arbais Village

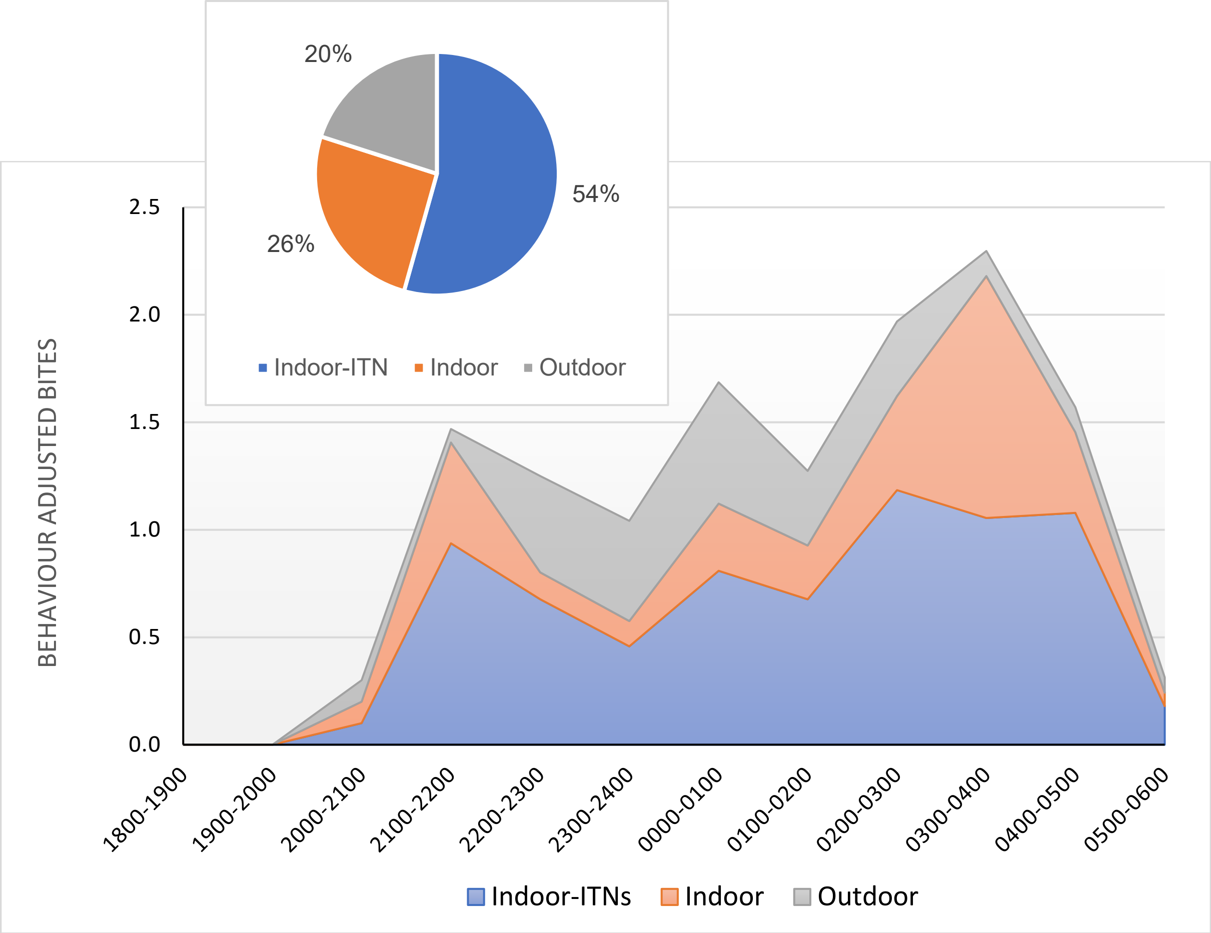


Figure S2-7c. Human Behavior Adjusted from Arbais Village

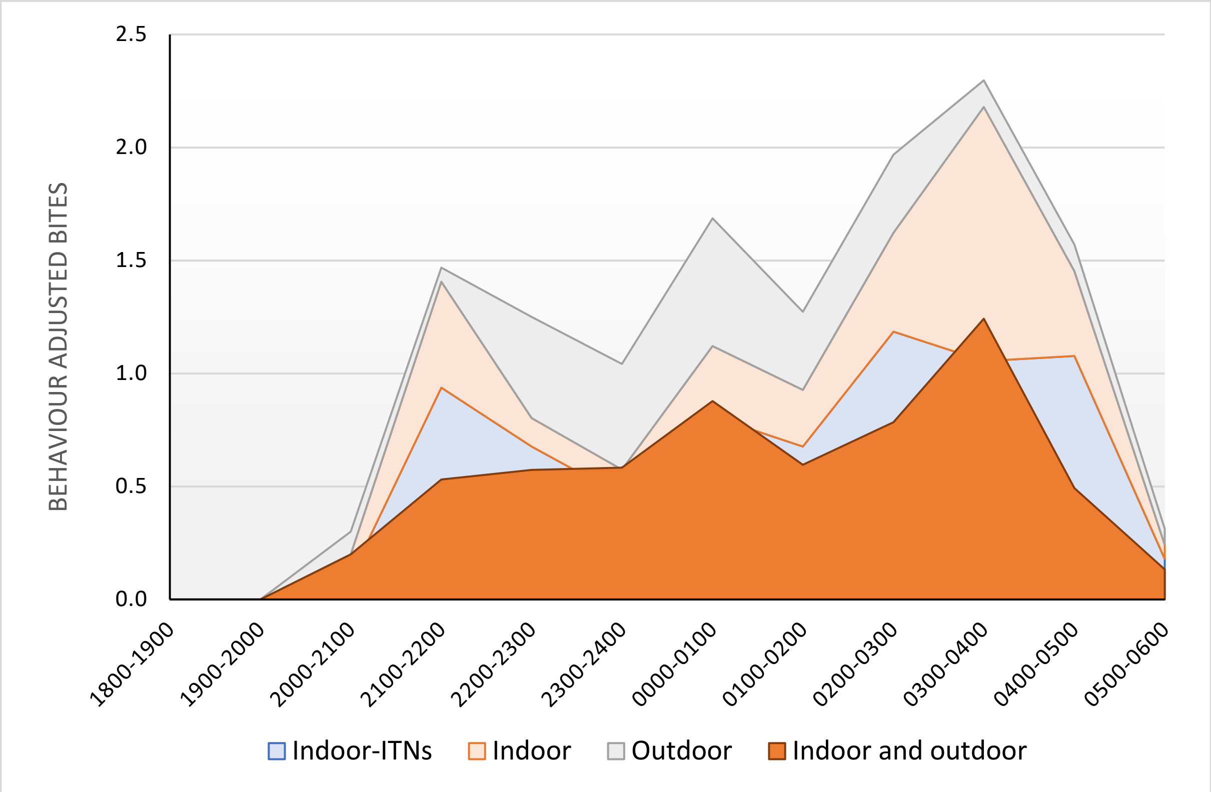


Figure S2-7d. Behavior-adjusted exposure rate for an unprotected individual from Arbais Village

8. Webrau Village, Sarmi Regency (Coastal, HBR = 23.0 bpn)

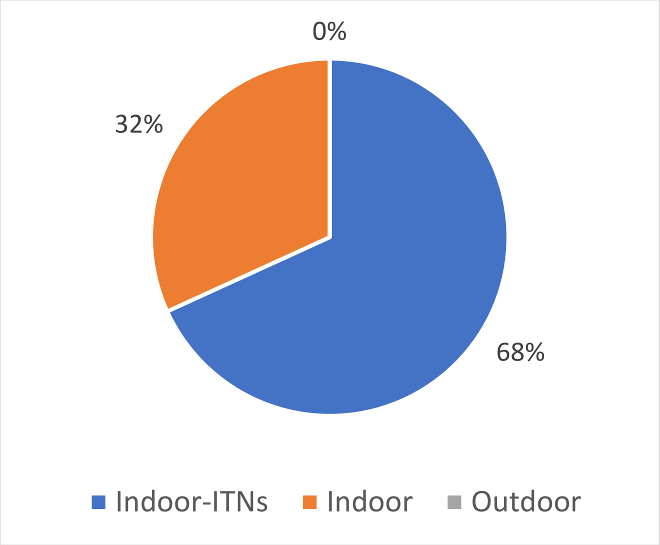


Figure S2-8a. Total human behavior proportion from Webrau Village

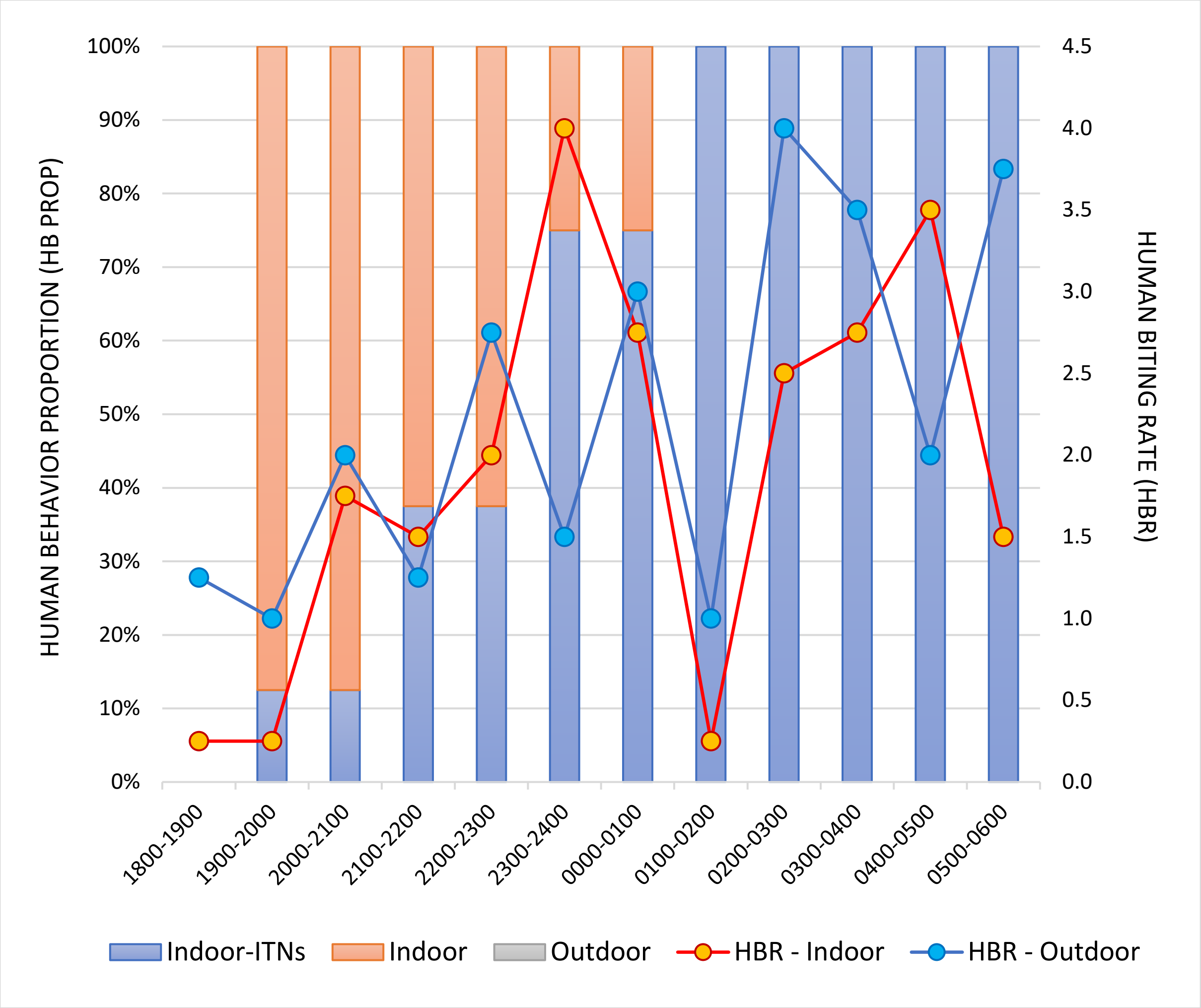


Figure S2-8b. Proportion of HBO vs indoor and outdoor HBR (bph) from Webrau Village

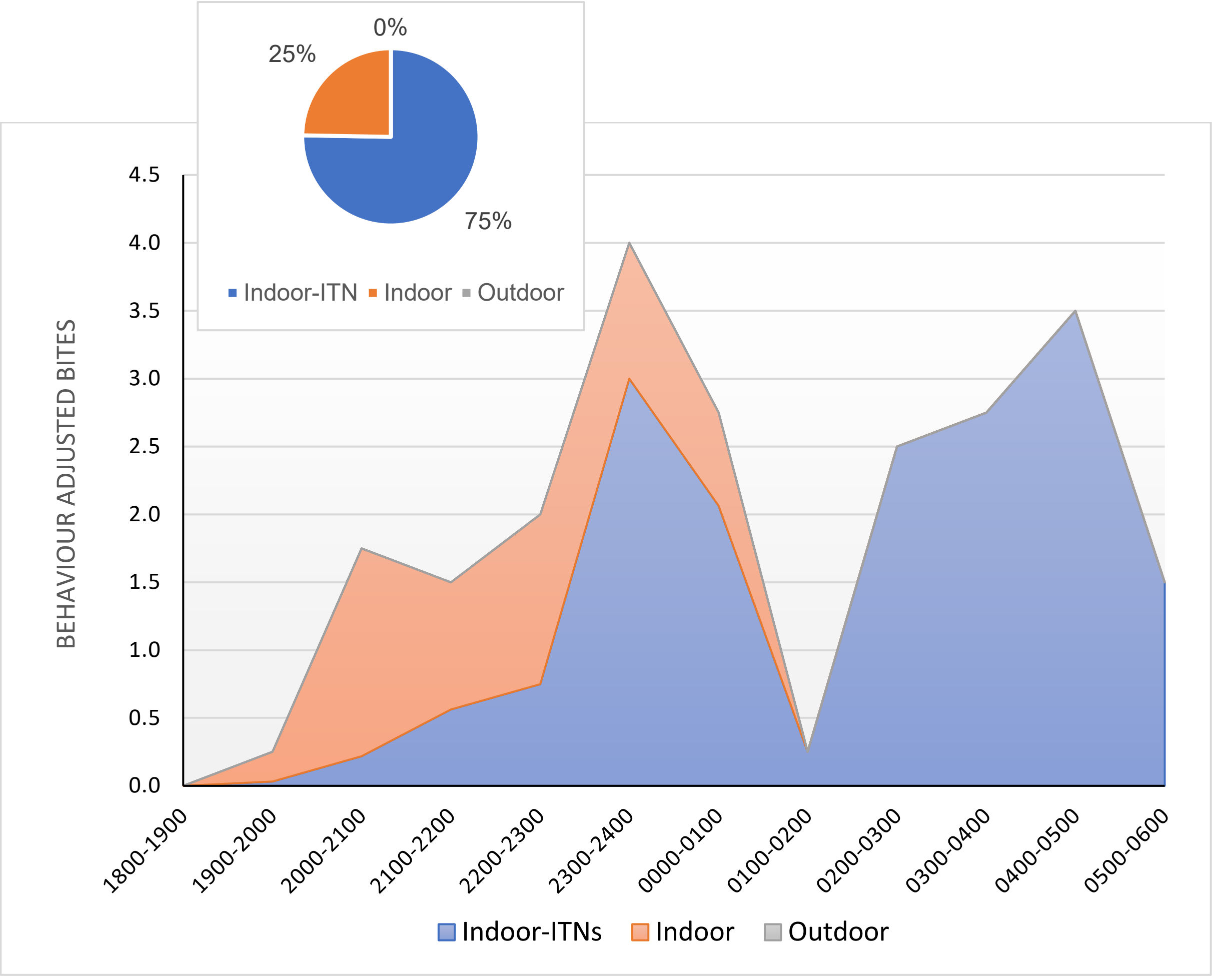


Figure S2-8c. Human Behavior Adjusted from Webrau Village

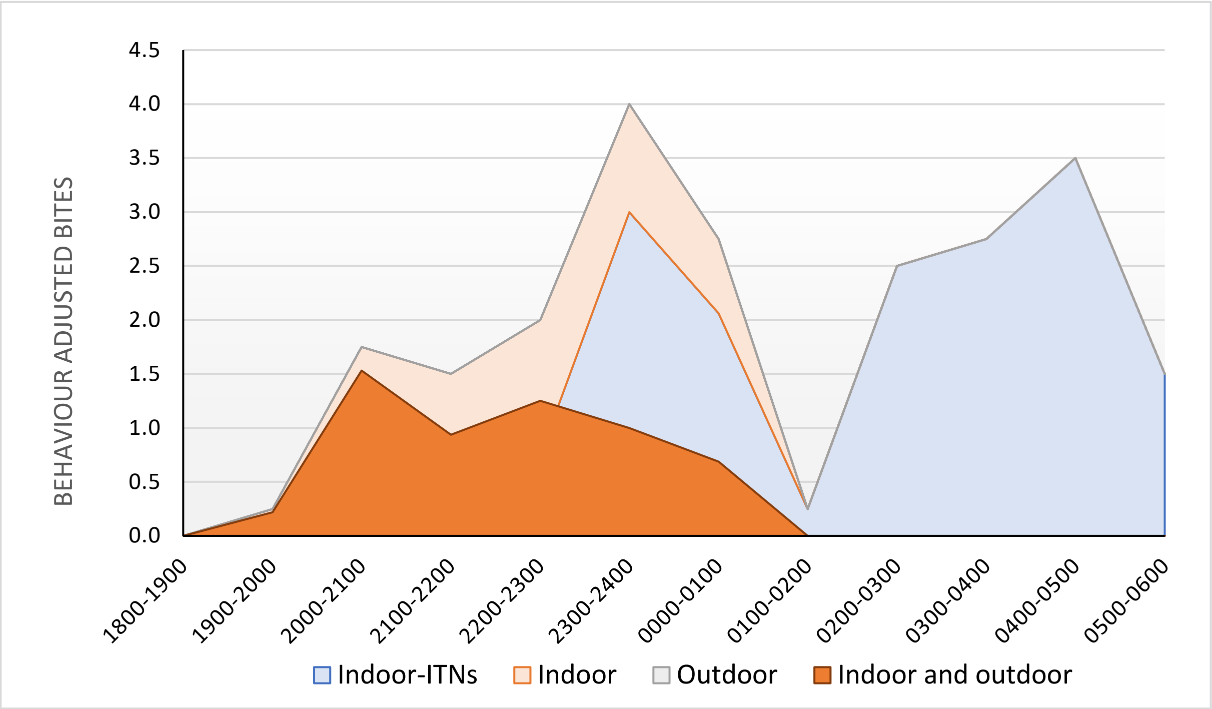


Figure S2-8d. Behavior-adjusted exposure rate for an unprotected individual from Webrau Village

9. Mwarei Village, Mimika Regency (Inland, HBR = 38.3 bpn)

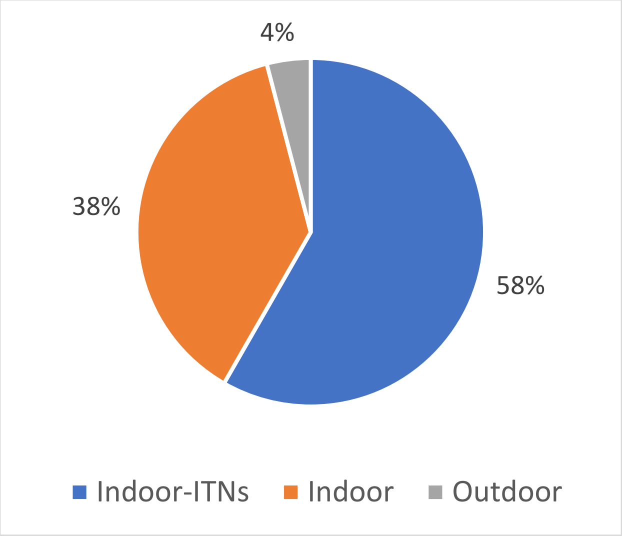


Figure S2-9a. Total human behavior proportion from Mwarei Village

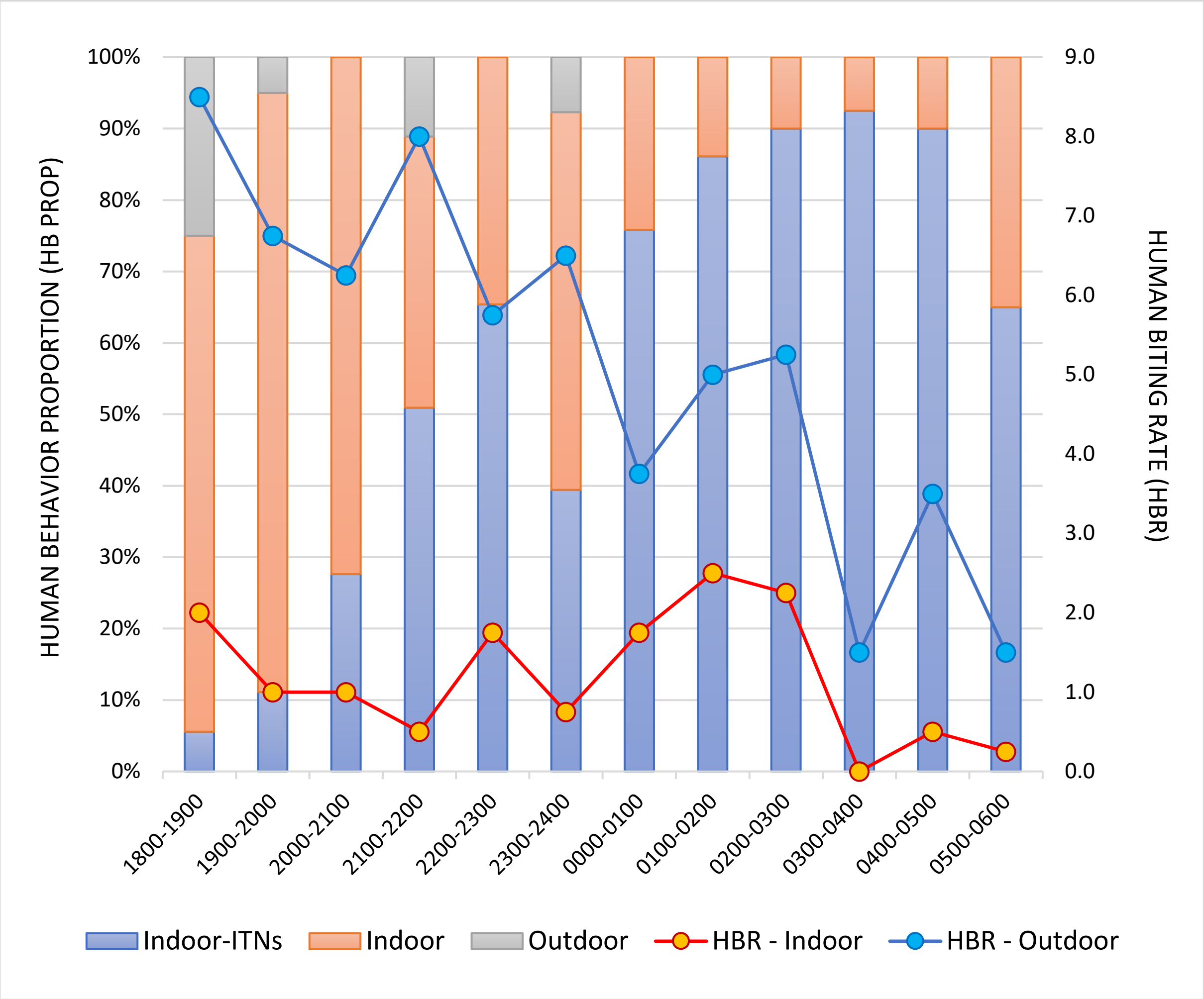


Figure S2-9b. Proportion of HBO vs indoor and outdoor HBR (bph) from Mwarei Village

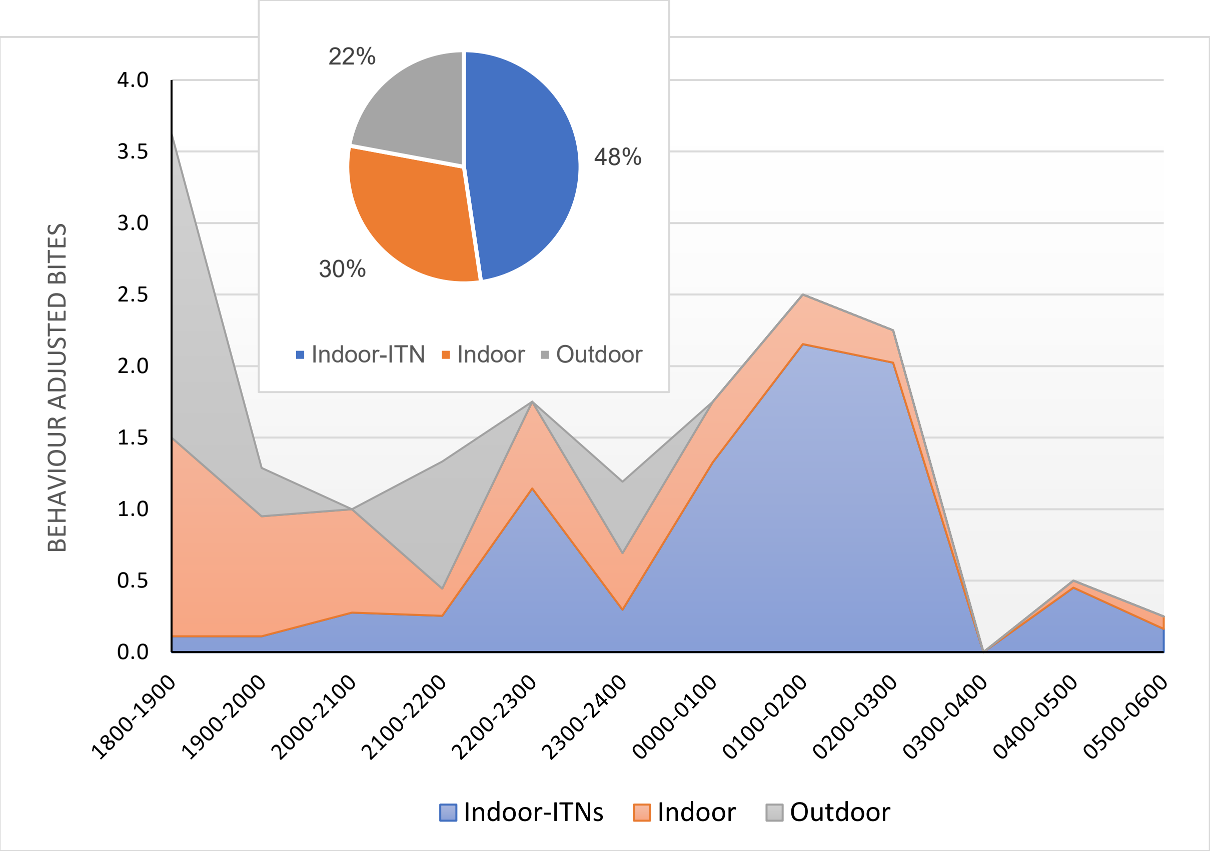


Figure S2-9c. Human Behavior Adjusted from Mwarei Village

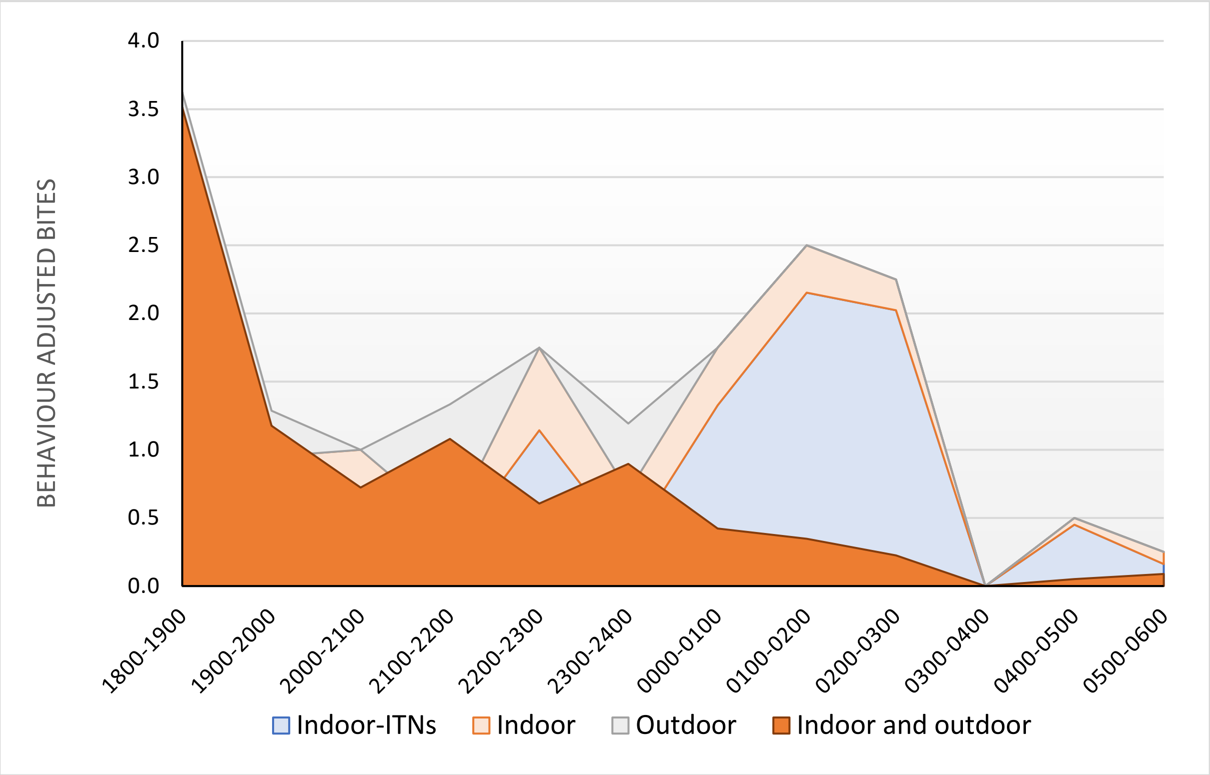


Figure S2-9d. Behavior-adjusted exposure rate for an unprotected individual from Mwarei Village

10. Kaugapu Village, Mimika Regency (Inland, HBR = 28.4 bpn)

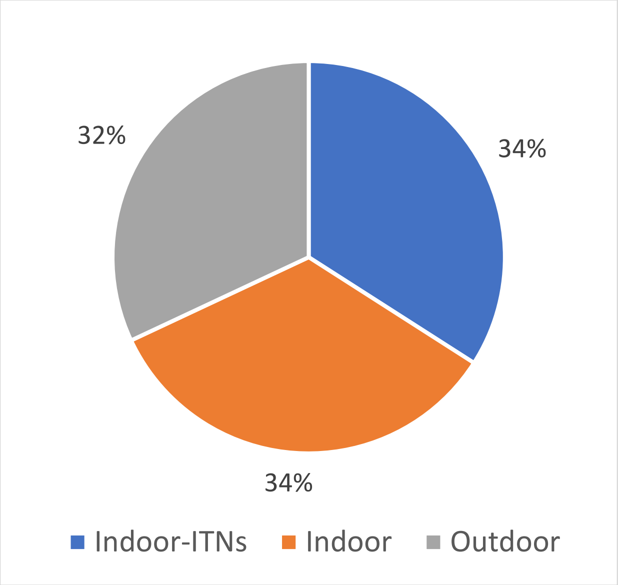


Figure S2-10a. Total human behavior proportion from Kaugapu Village

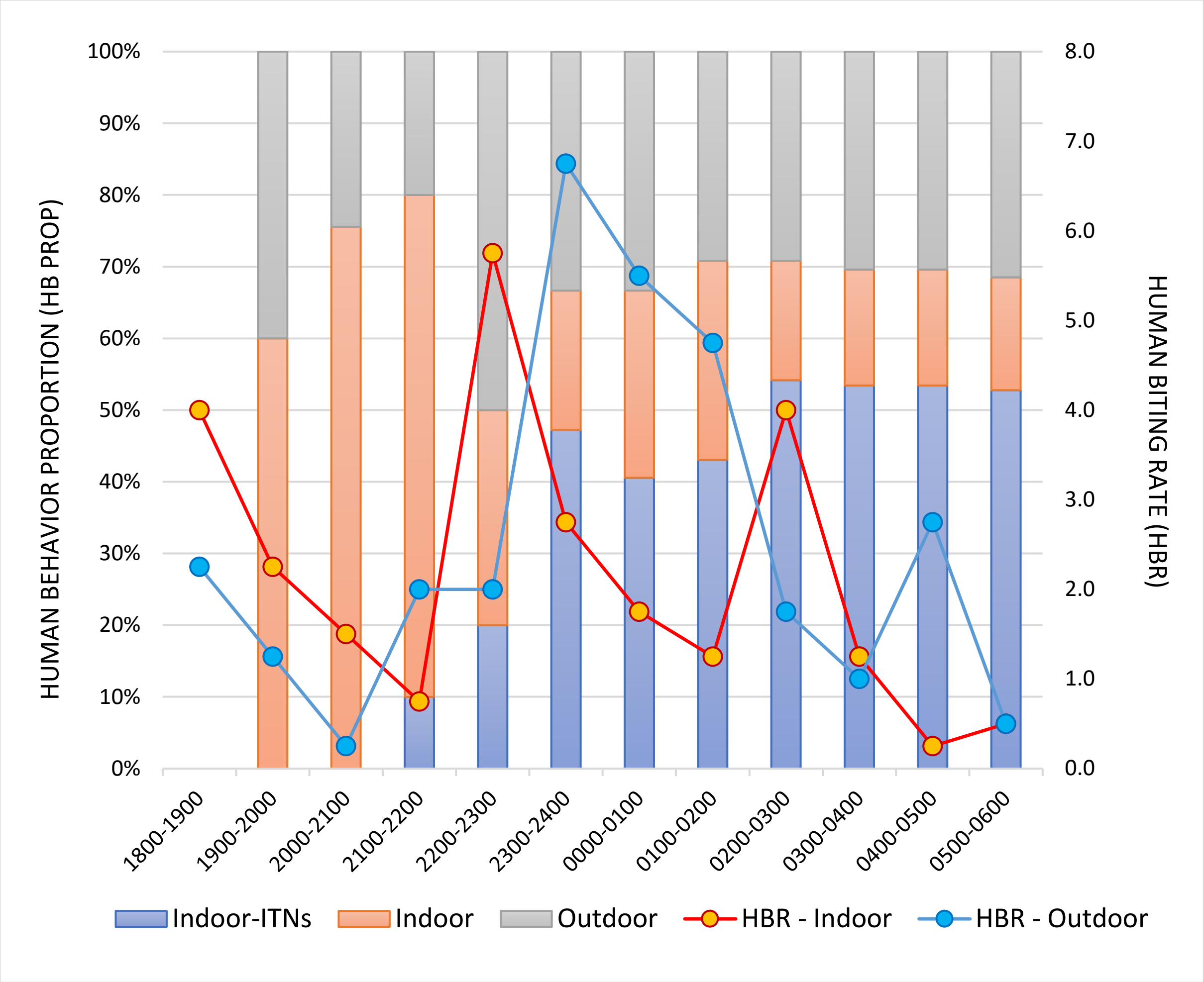


Figure S2-10b. Proportion of HBO vs indoor and outdoor HBR (bph) from Kaugapu Village

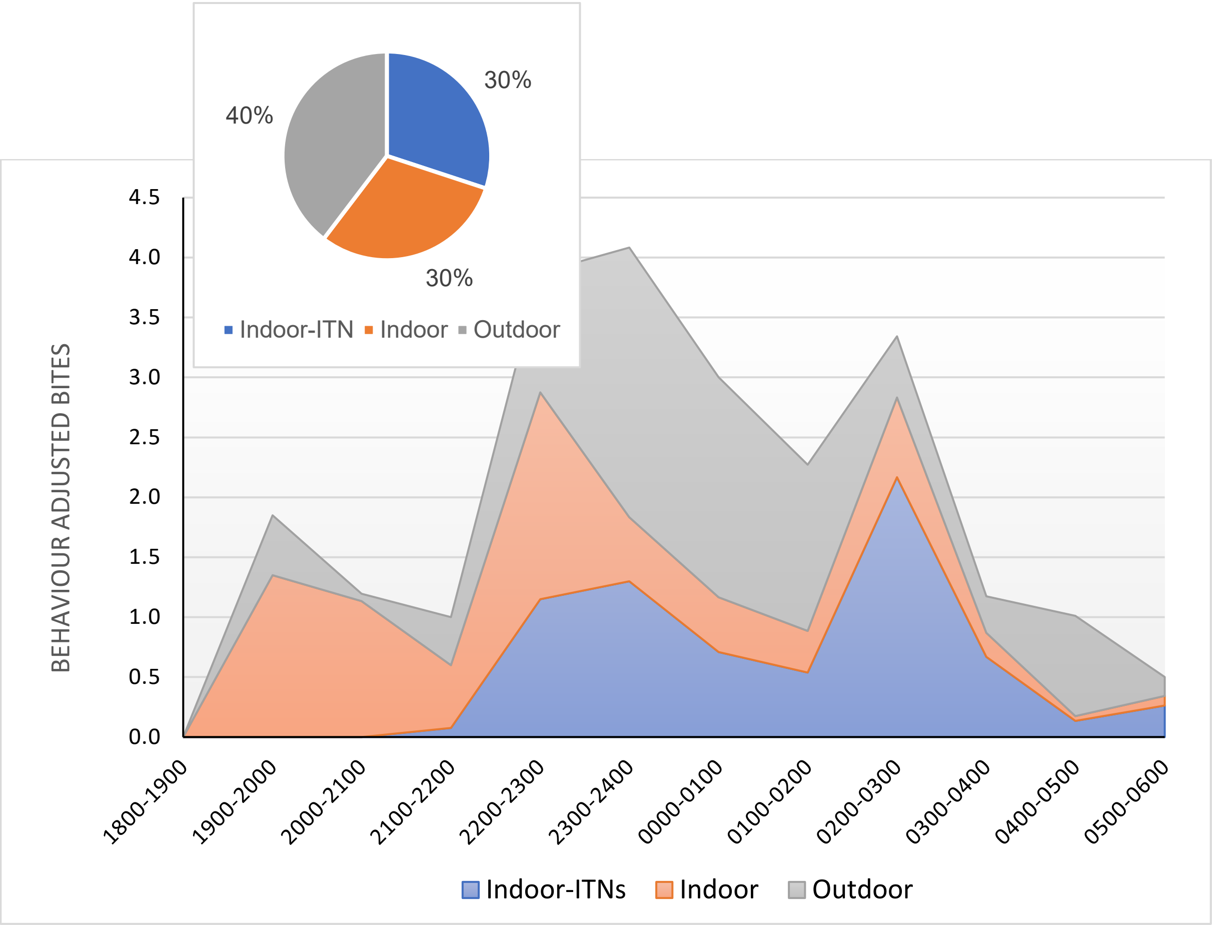


Figure S2-10c. Human Behavior Adjusted from Kaugapu Village

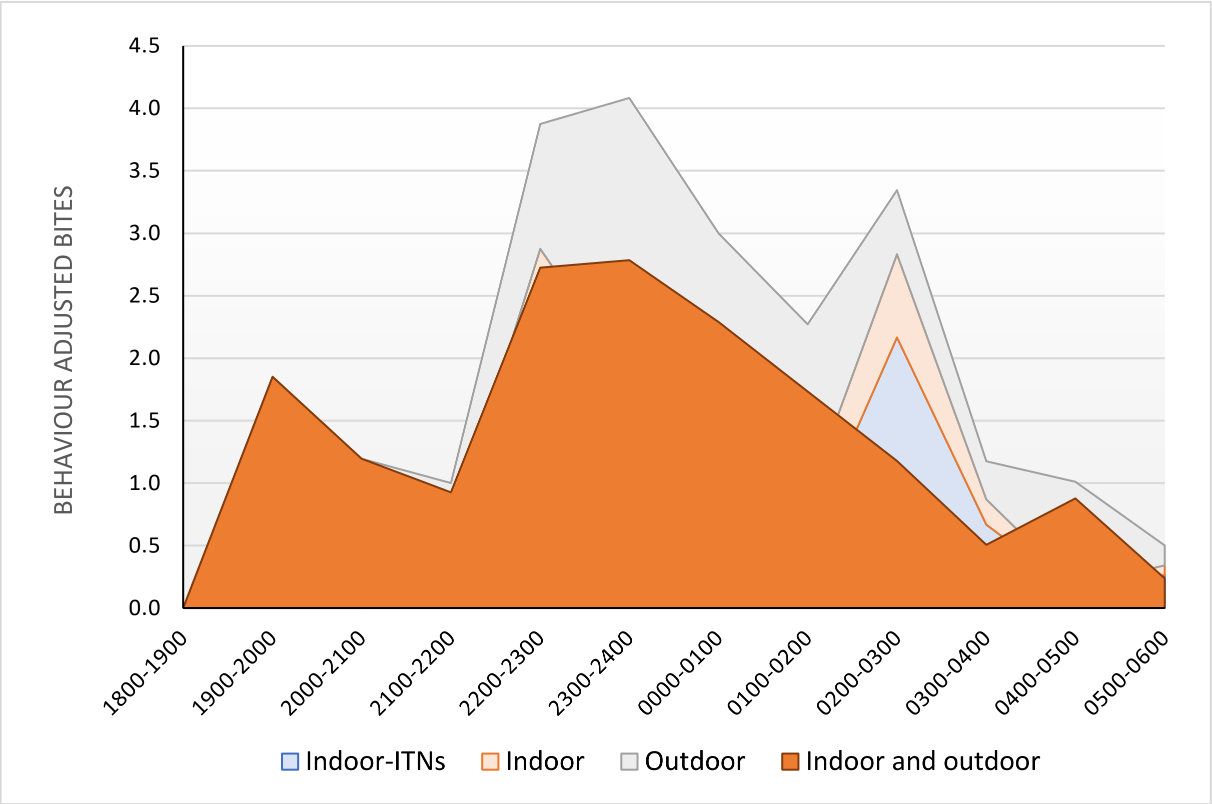


Figure S2-10d. Behavior-adjusted exposure rate for an unprotected individual from Kaugapu Village

11. Wanagon Village, Mimika Regency (Inland, HBR = 12.9 bpn)

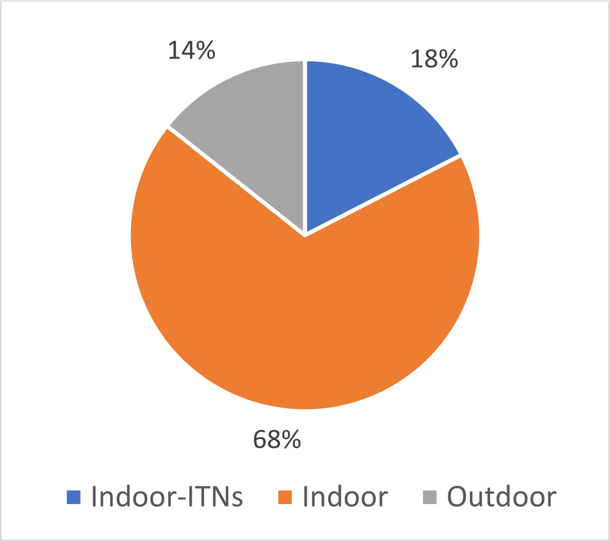


Figure S2-11a. Total human behavior proportion from Wanagon Village

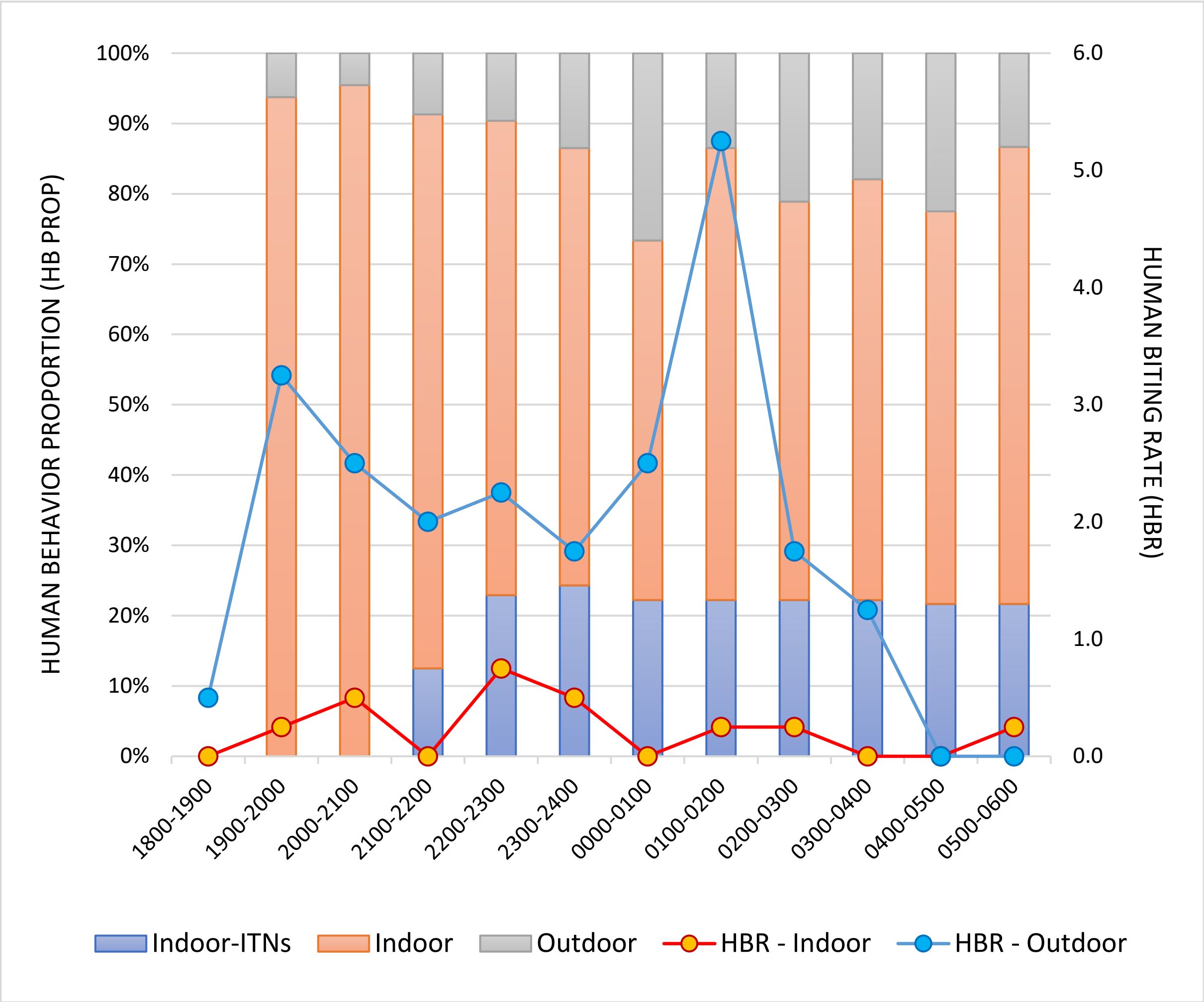


Figure S2-11b. Proportion of HBO vs indoor and outdoor HBR (bph) from Wanagon Village

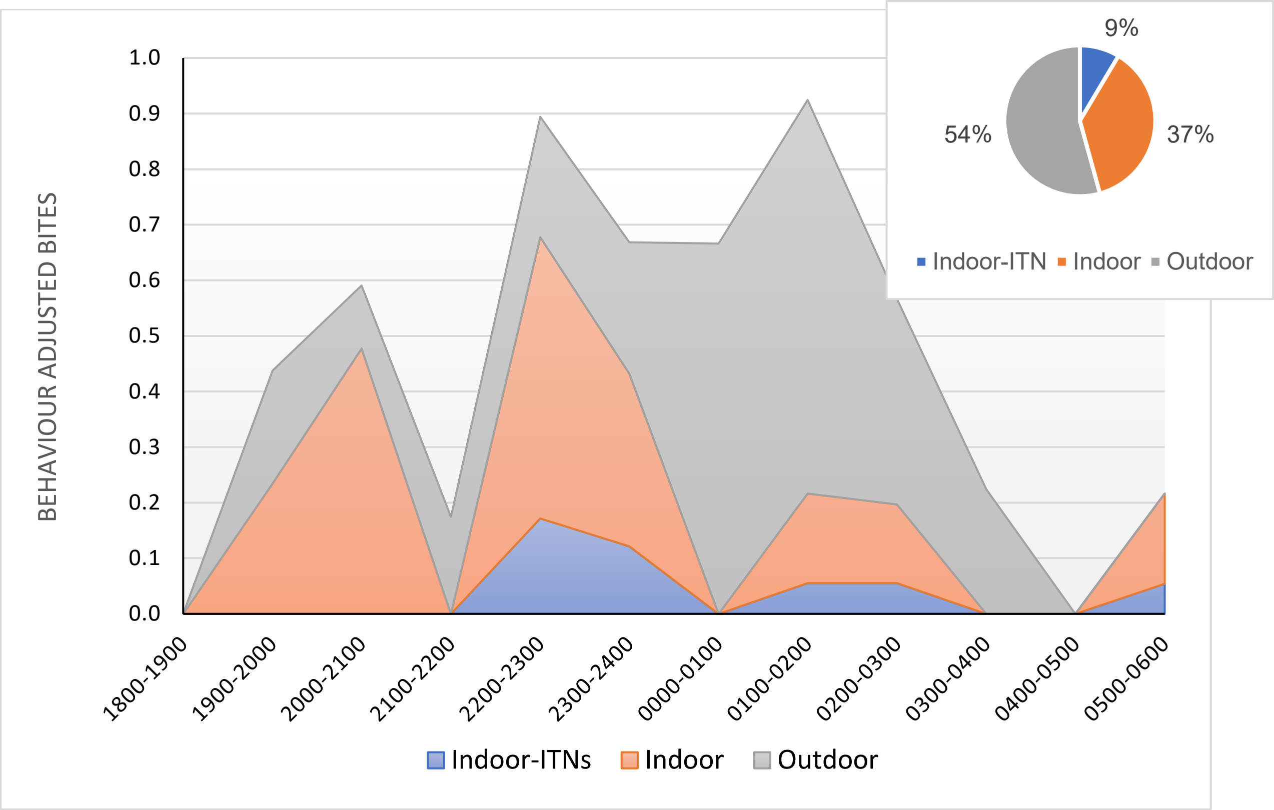


Figure S2-11c. Human Behavior Adjusted from Wanagon Village

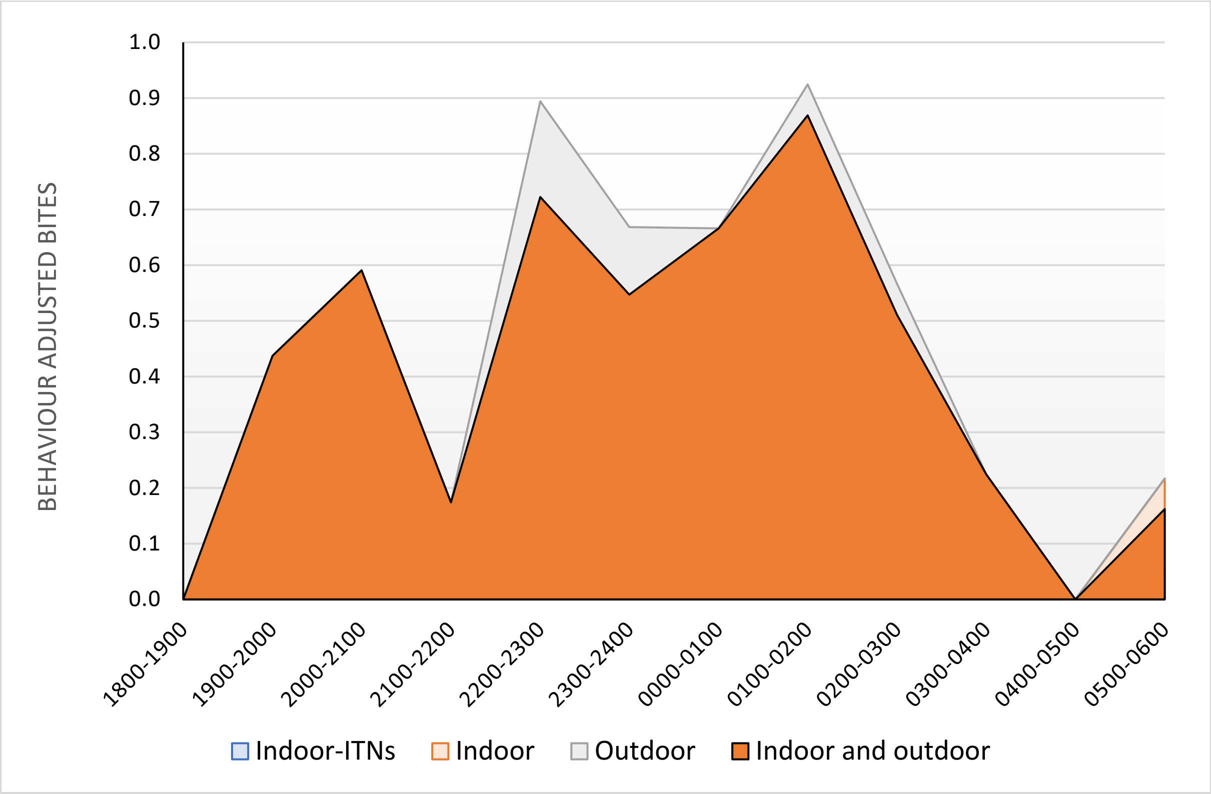


Figure S2-11d. Behavior-adjusted exposure rate for an unprotected individual from Wanagon Village

12. Sukanggo Village, Boven Digoel Regency (Inland, HBR = 10.3 bpn)

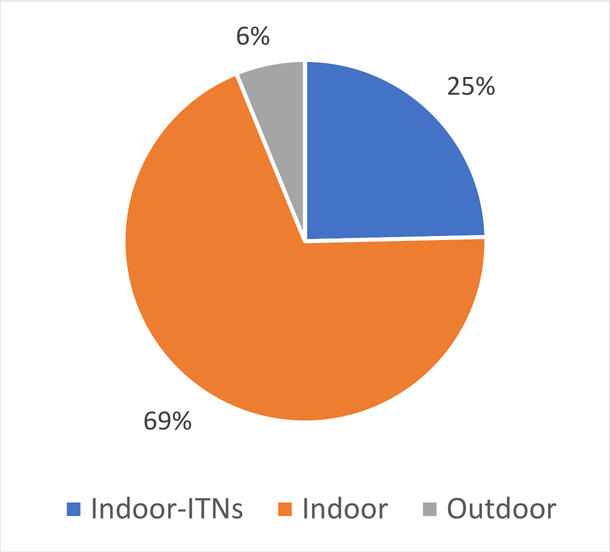


Figure S2-12a. Total human behavior proportion from Sukanggo Village

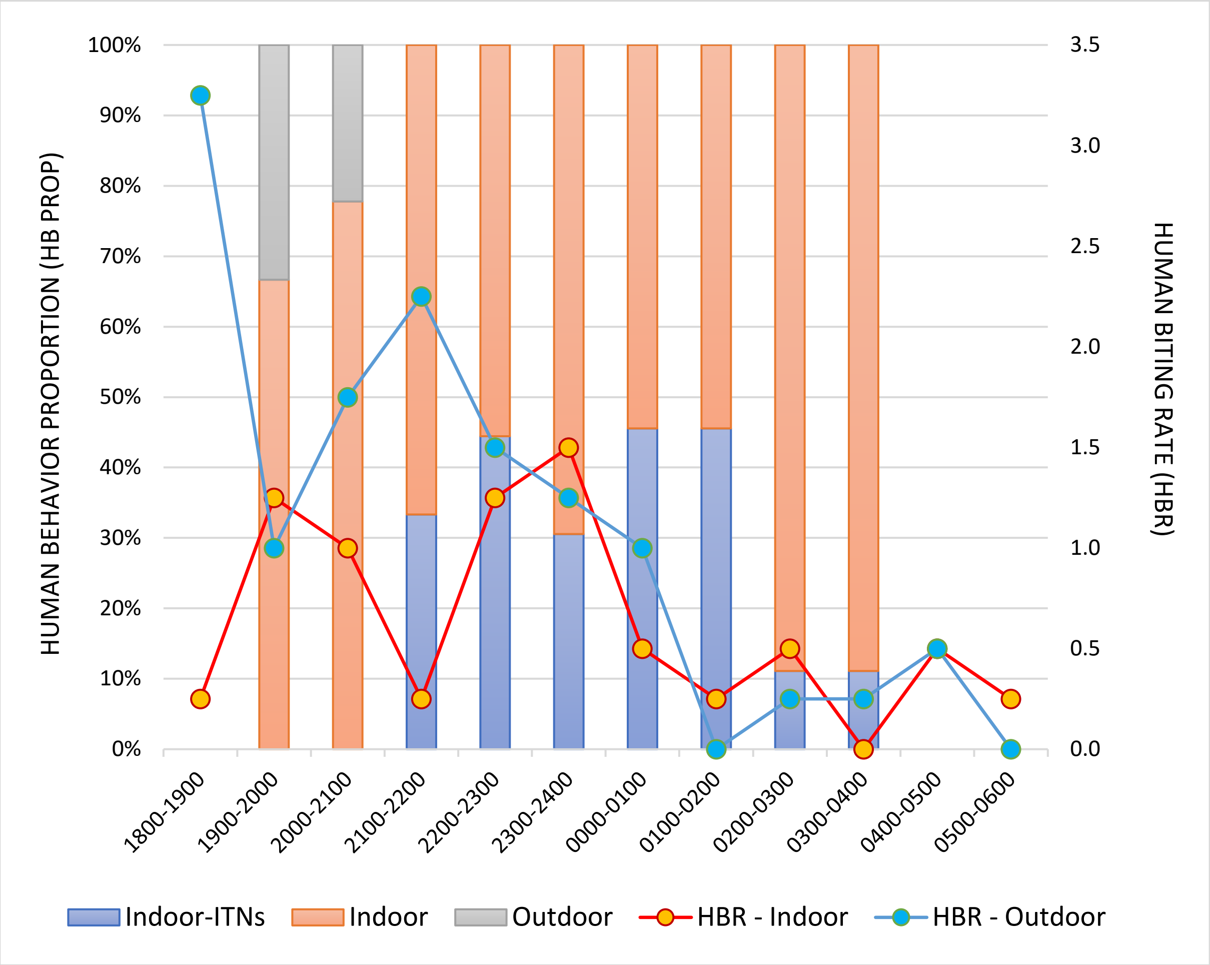


Figure S2-12b. Proportion of HBO vs indoor and outdoor HBR (bph) from Sukanggo Village

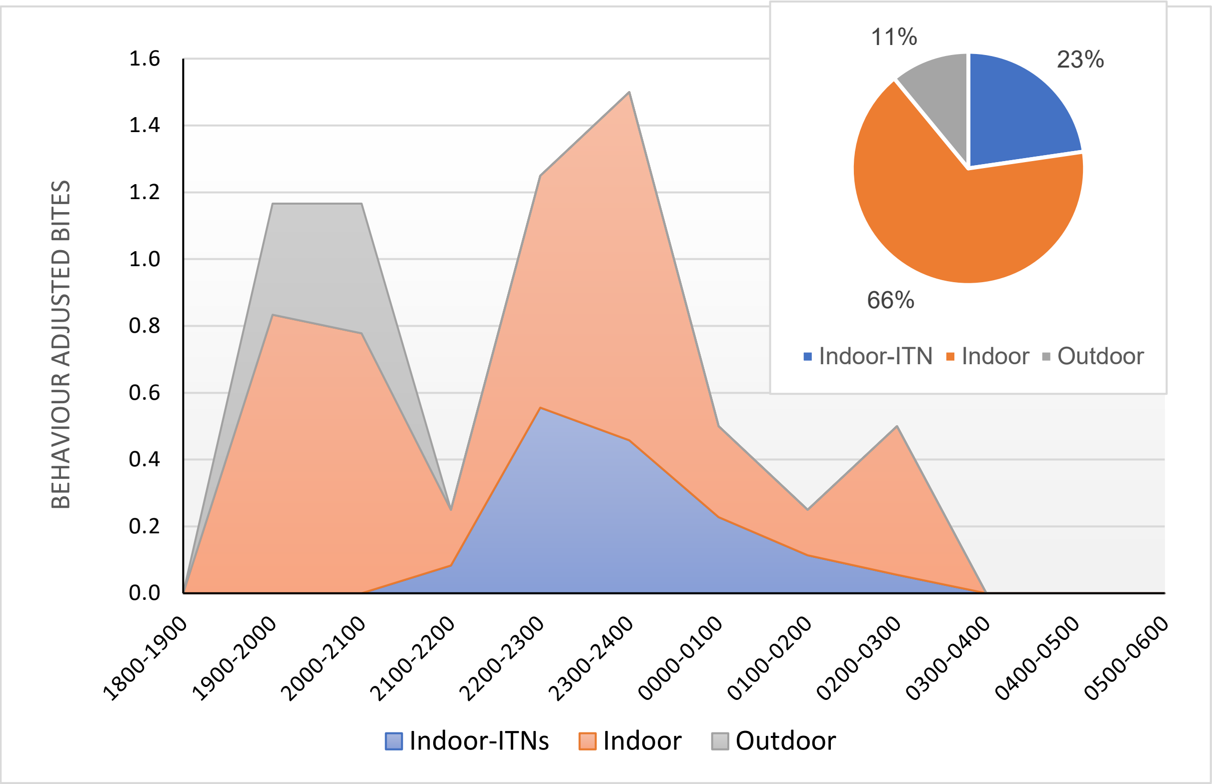


Figure S2-12c. Human Behavior Adjusted from Sukanggo Village

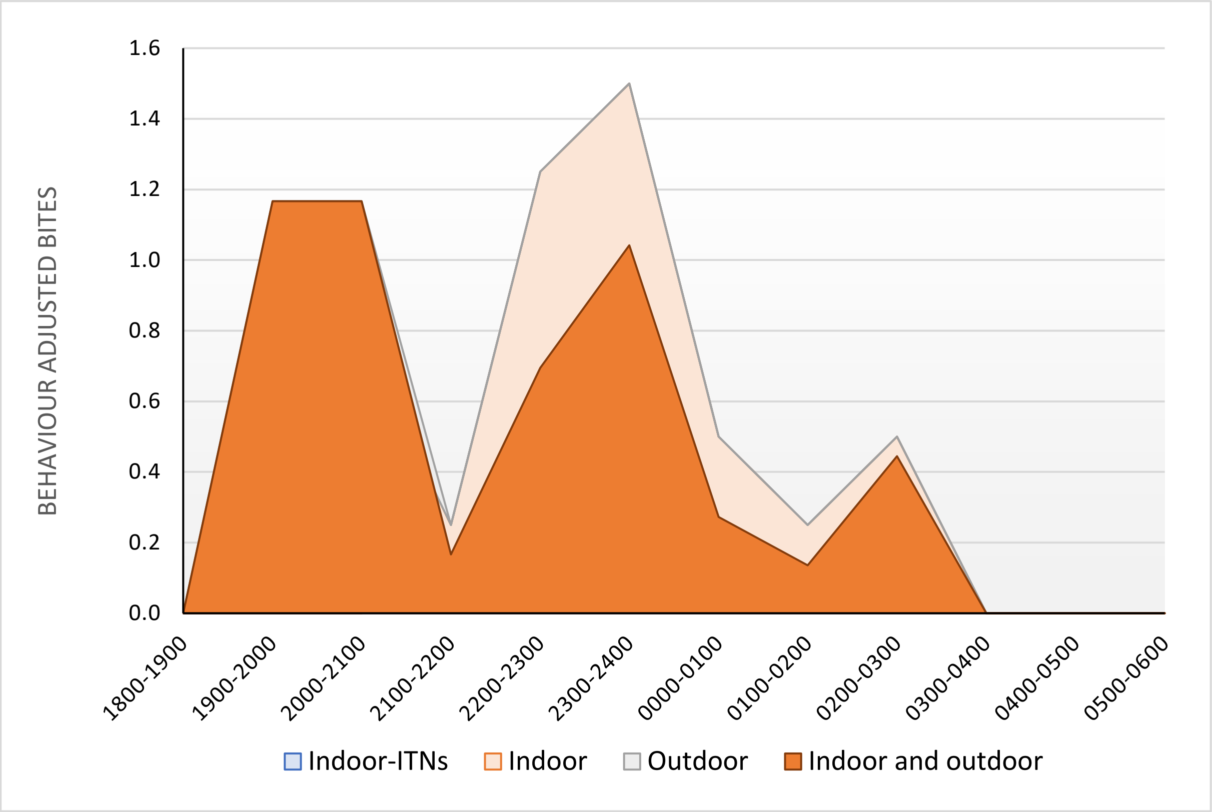


Figure S2-12d. Behavior-adjusted exposure rate for an unprotected individual from Sukanggo Village

13. Migiwia Village, Mimika Regency (Coastal, HBR = 7.25 bpn)

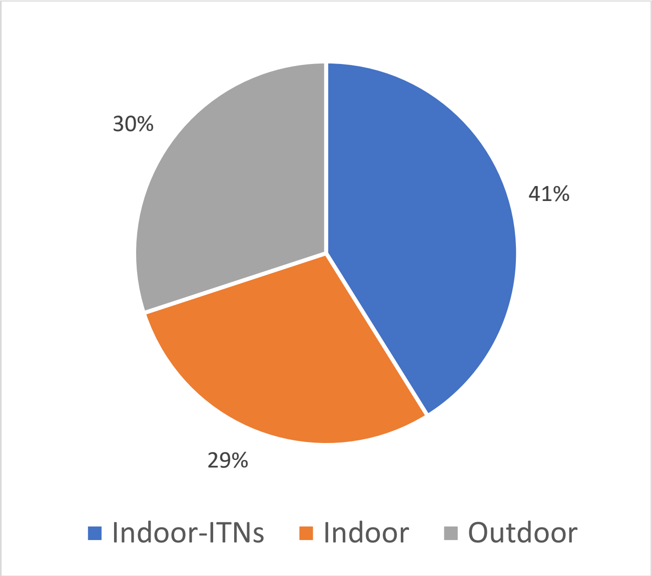


Figure S2-13a. Total human behavior proportion from Migiwia Village

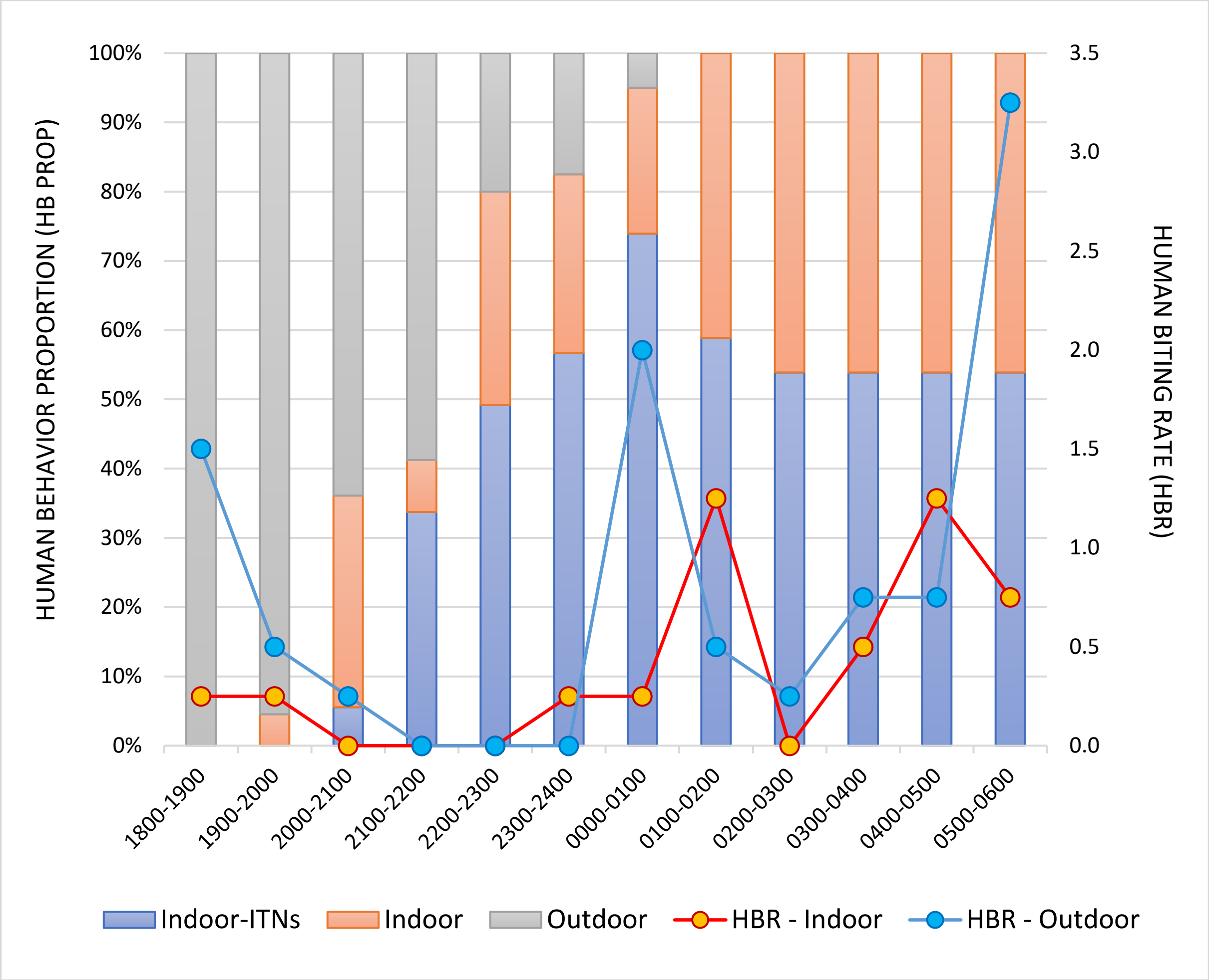


Figure S2-13b. Proportion of HBO vs indoor and outdoor HBR (bph) from Migiwia Village

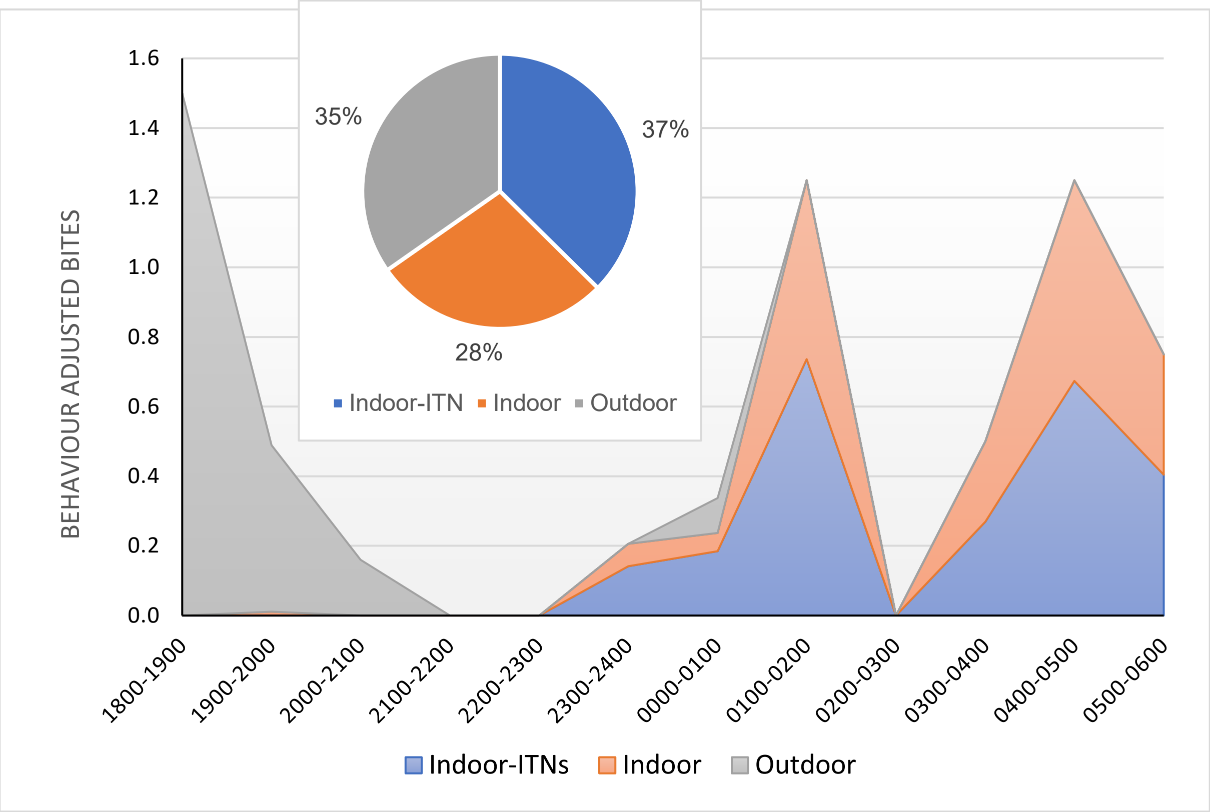


Figure S2-13c. Human Behavior Adjusted from Migiwia Village

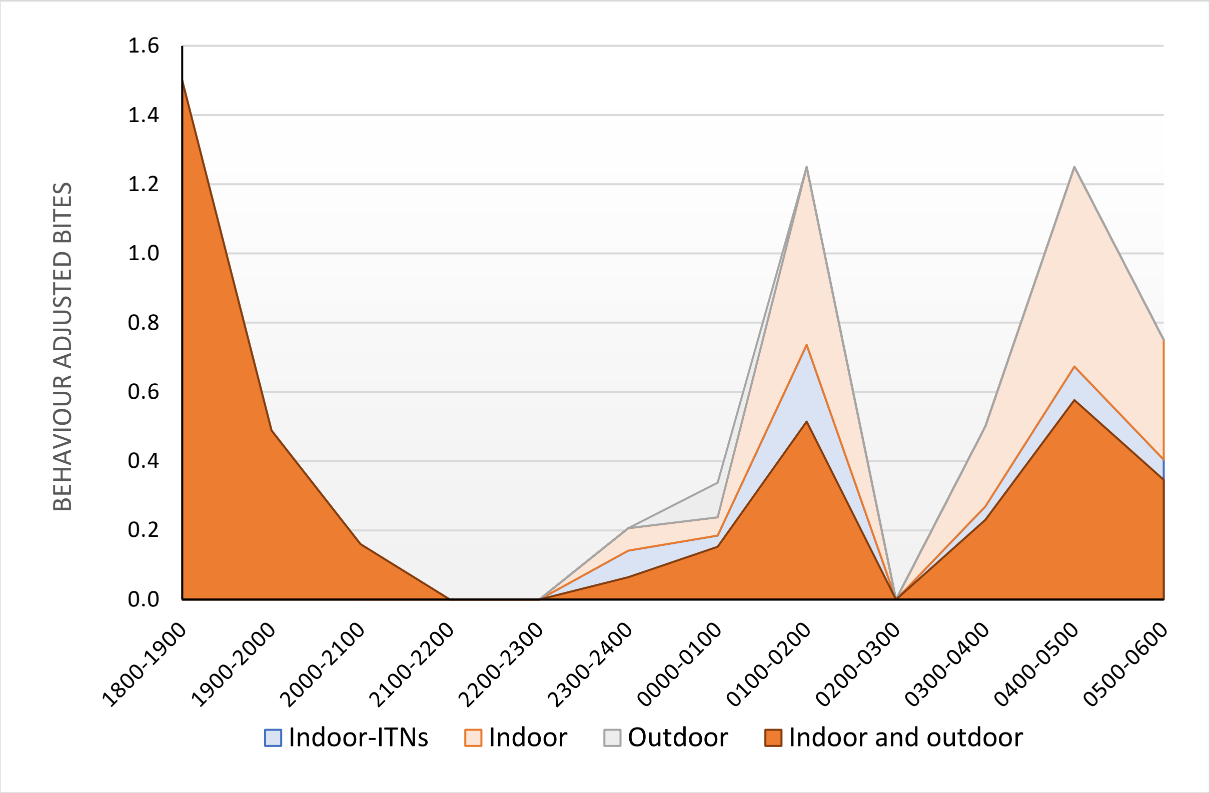


Figure S2-13d. Behavior-adjusted exposure rate for an unprotected individual from Migiwia Village

14. Yasiuw Village, Asmat Regency (River side, HBR = 7.00 bpn)

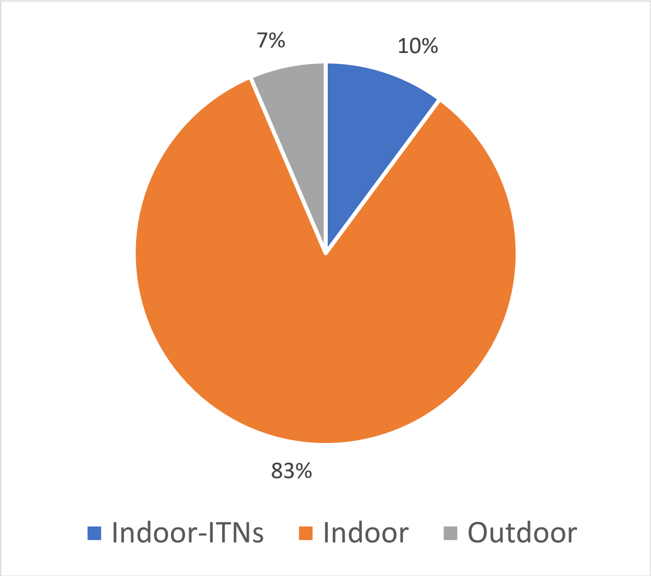


Figure S2-14a. Total human behavior proportion from Yasiuw Village

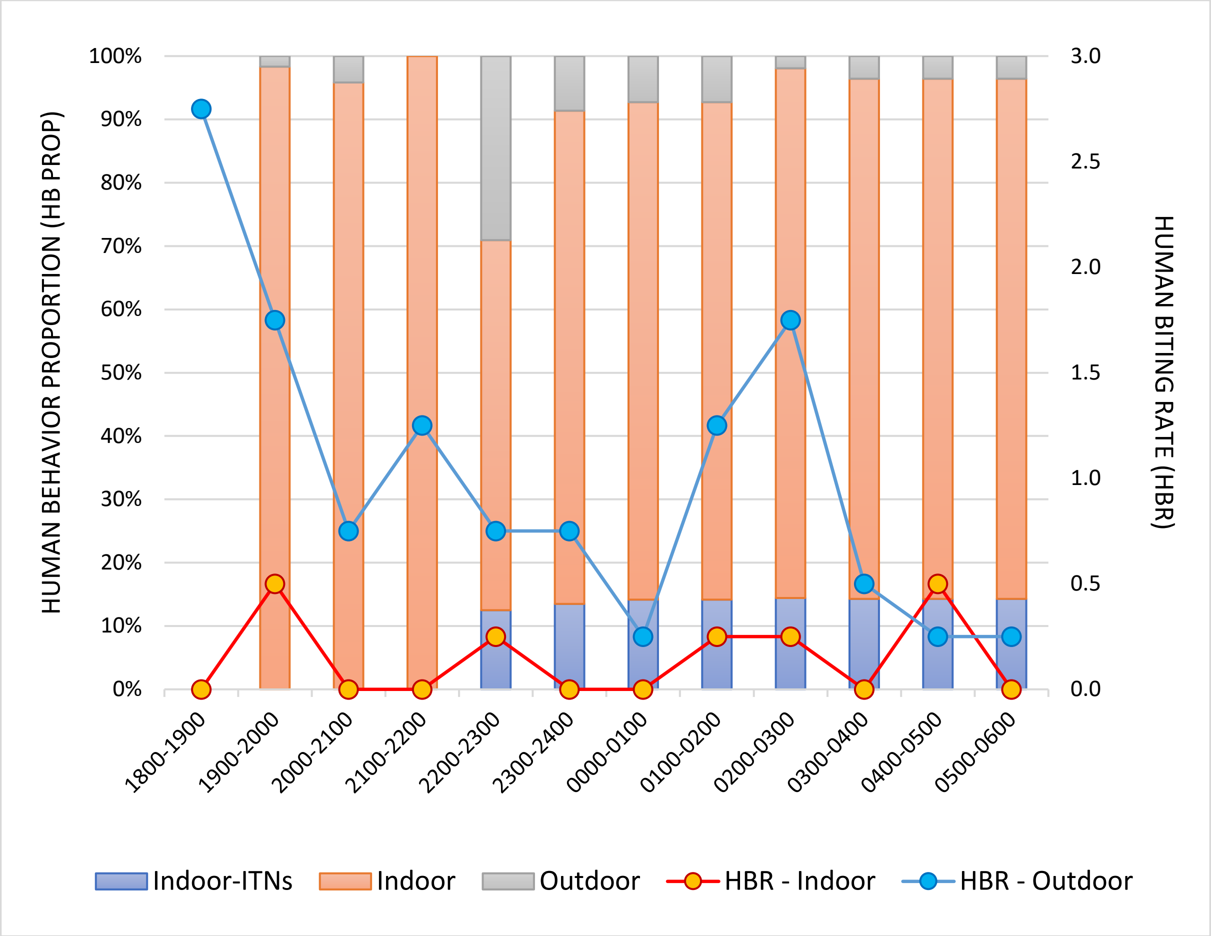


Figure S2-14b. Proportion of HBO vs indoor and outdoor HBR (bph) from Yasiuw Village

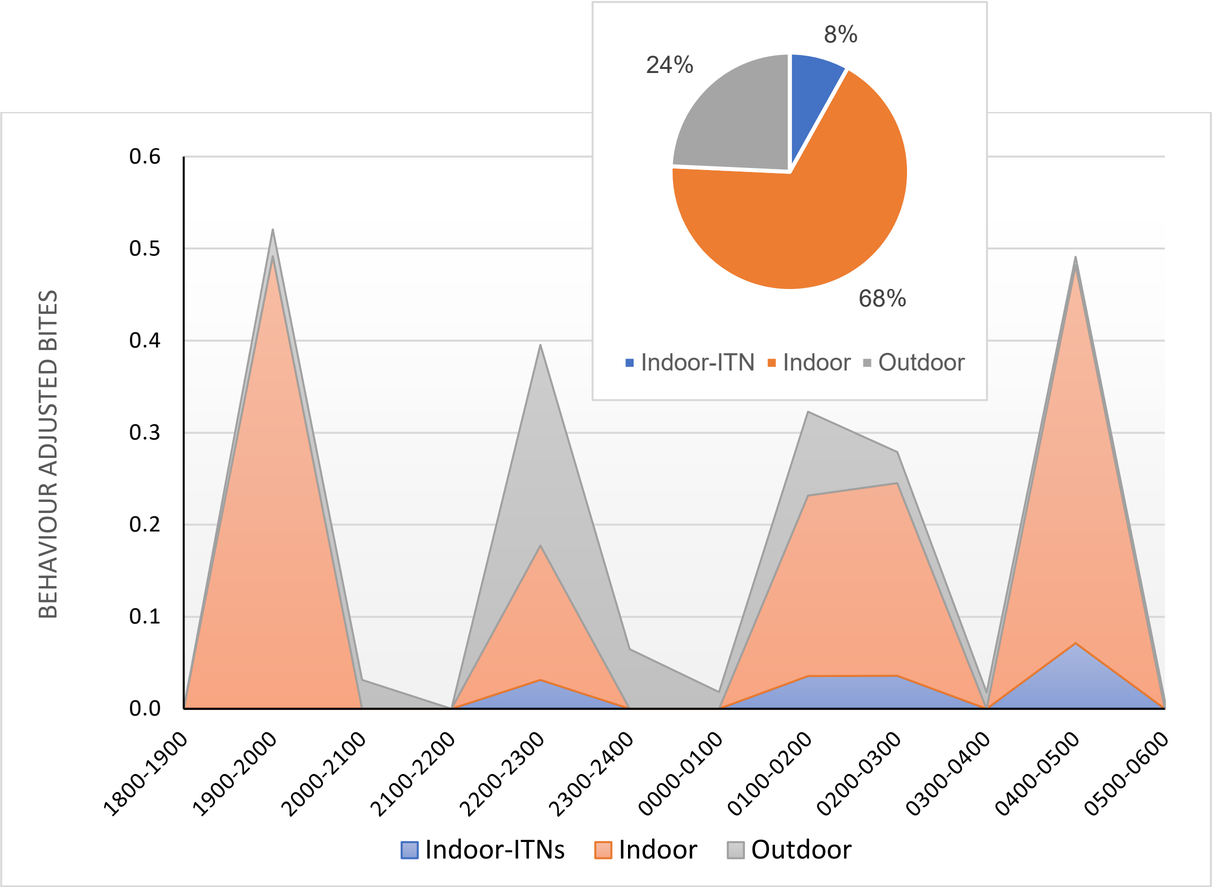


Figure S2-14c. Human Behavior Adjusted from Yasiuw Village

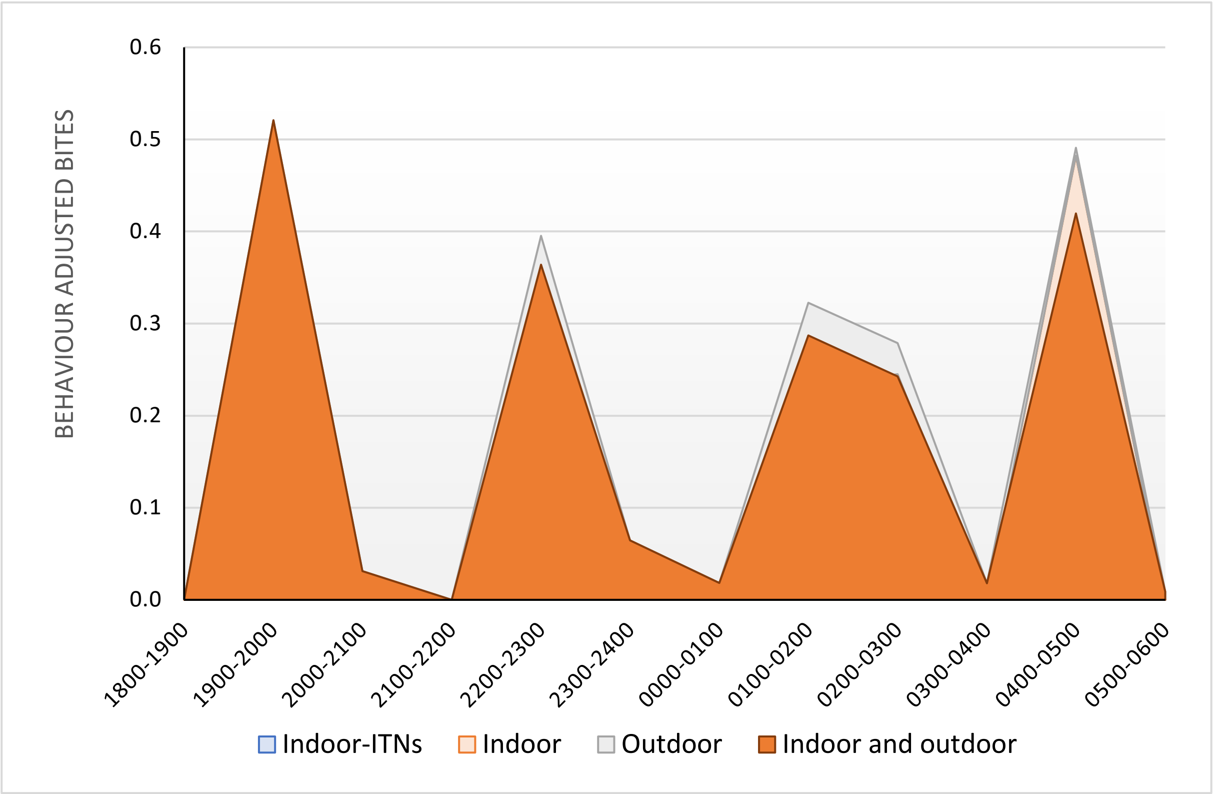


Figure S2-14d. Behavior-adjusted exposure rate for an unprotected individual from Yasiuw Village
