# Supplementary material for: Gaps in protection to Anopheles exposure in high malaria endemic regencies of Papua Province, Indonesia
Source: PLoS One. 2025 Apr 11;20(4):e0311076. doi: 10.1371/journal.pone.0311076 (PMC11990486; doi:10.1371/journal.pone.0311076)
Supplement: S3 File — (PDF) [file pone.0311076.s003.pdf]

Supplementary File 3

Household survey results

Table S3-1. The interviewee's data

Interviewee's data	Keerom n=168	Jayapura n=179	Sarmi n=158	Mimika n=168	Boven Digoel n=142	Yapen Islands n=101	Waropen n=180	Asmat n=155	Total n=1251
**Gender**									
Male	78 (46.4%)	78 (43.6%)	90 (57.0%)	59 (35.1%)	75 (52.8%)	57 (56.4%)	129 (71.7%)	97 (62.6%)	663 (53.0%)
Female	90 (53.6%)	101 (56.4%)	68 (43.0%)	109 (64.9%)	67 (47.2%)	44 (43.6%)	51 (28.3%)	58 (37.4%)	588 (47.0%)
**Relationship**									
Head of household (or his/her spouse)	86.31%	88.27%	82.91%	79.76%	87.32%	87.13%	95.00%	84.52%	86.49%
Other primary family	11.31%	11.73%	13.92%	19.05%	11.97%	9.90%	4.44%	14.84%	12.15%
Not primary family	2.38%	0.00%	3.16%	1.19%	0.70%	2.97%	0.56%	0.65%	1.36%
**Age (years)**									
Average (SD)	38.2 (± 13.2)	45.0 (± 14.9)	40.8 (± 13.3)	38.0 (± 14.6)	40.1 (± 13.5)	41.2 (± 14.3)	43.7 (± 13.3)	38.5 (± 13.2)	40.8 (± 14.0)
Range	16 - 73	15 - 80	15 - 82	15 - 78	15 - 80	18 - 74	18 - 83	15 - 78	15 - 83
**Last Education**									
No education	8.9%	4.5%	7.0%	6.5%	9.2%	0.0%	2.2%	9.0%	6.1%
Not finish primary school	3.6%	7.8%	5.7%	3.6%	7.0%	2.0%	3.3%	22.6%	7.0%
Primary or Junior high	44.6%	50.3%	55.7%	54.2%	33.8%	45.5%	47.8%	40.6%	46.9%
Senior high or above	42.9%	37.4%	31.0%	35.1%	49.3%	52.5%	46.7%	27.1%	39.6%

Table S3-2. The household's structures.

Household based data	Keerom n=168	Jayapura n=179	Sarmi n=158	Mimika n=168	Boven Digoel n=142	Yapen Islands n=101	Waropen n=180	Asmat n=155	Total n=1251
**Total Households**									
Average per village	28.0 (± 6.5)	29.8 (± .4)	26.3 (± 4.6)	28.0 (± 2.5)	23.7 (± 2.7)	20.2 (± 1.1)	30.0 (± 5.5)	25.8 (± 3.2)	26.6 (± 4.7)
Total household members average	4.3 (± 2.0)	5.0 (± 2.9)	6.3 (± 5.4)	5.8 (± 2.5)	5.8 (± 3.0)	6.2 (± 2.9)	4.5 (± 2.3)	7.2 (± 4.5)	5.6 (± 3.5)
**Socioeconomic status**									
1st Quantile	14.9%	16.8%	34.2%	29.2%	54.9%	19.8%	11.7%	29.0%	25.7%
2nd Quantile	45.2%	33.5%	50.0%	45.2%	18.3%	42.6%	43.3%	54.2%	41.7%
3rd Quantile	13.1%	22.3%	10.8%	13.1%	7.0%	16.8%	17.8%	9.0%	13.9%
4th Quantile	19.0%	20.1%	3.2%	8.9%	17.6%	18.8%	22.8%	3.9%	14.3%
5th Quantile	7.7%	7.3%	1.9%	3.6%	2.1%	2.0%	4.4%	3.9%	4.3%
**House type**									
Type 1	28.0%	27.9%	63.9%	44.6%	14.1%	38.6%	30.0%	99.4%	43.2%
Type 2	44.6%	3.9%	10.8%	15.5%	30.3%	13.9%	16.1%	0.0%	16.9%
Type 3	22.0%	48.6%	24.7%	35.7%	35.2%	43.6%	28.9%	0.0%	29.5%
Type 4	5.4%	19.6%	0.6%	4.2%	20.4%	4.0%	25.0%	0.6%	10.5%
**Wall material**									
Wooden plank / half cement half wooden plank	64.88%	49.72%	66.46%	49.40%	48.59%	48.51%	60.56%	96.77%	60.99%
Cement / concrete / stone	35.12%	50.28%	29.11%	50.00%	50.70%	51.49%	35.56%	0.00%	37.33%
Others	0.00%	0.00%	4.43%	0.60%	0.70%	0.00%	3.89%	3.23%	1.68%
**Roof material**									
Metal (zinc sheeting)	94.64%	88.83%	97.47%	98.21%	88.03%	93.07%	99.44%	83.23%	93.05%
Others	5.36%	11.17%	2.53%	1.79%	11.97%	6.93%	0.56%	16.77%	6.95%

**Floor material**									
Wooden plank	27.98%	27.37%	61.39%	44.64%	14.08%	38.61%	26.67%	96.13%	41.89%
Ceramic	12.50%	20.67%	6.96%	29.17%	15.49%	16.83%	25.00%	0.00%	16.15%
Cement	59.52%	49.72%	29.11%	26.19%	69.01%	44.55%	43.89%	0.00%	40.05%
Others	0.00%	2.23%	2.53%	0.00%	1.41%	0.00%	4.44%	3.87%	1.92%
**Floor height**									
**• Wooden board floor**									
Groundlevel to 50 cm	5.95%	18.99%	20.25%	2.98%	4.23%	15.84%	5.56%	0.00%	9.03%
More than 50 cm	22.02%	8.38%	41.14%	41.67%	9.86%	22.77%	21.11%	96.13%	32.85%
**• Other floors**									
Groundlevel to 50 cm	61.90%	71.51%	29.75%	51.79%	76.76%	28.71%	35.56%	0.00%	45.40%
More than 50 cm	10.12%	1.12%	8.86%	3.57%	9.15%	32.67%	37.78%	3.87%	12.71%
**Door**									
Average	2.4 (± 0.7)	2.6 (± 0.8)	2.1 (± 0.6)	2.3 (± 0.7)	2.2 (± 0.9)	2.9 (± 0.9)	2.7 (± 0.9)	2.3 (± 0.8)	2.4 (± 0.8)
Average screened door	0.0	0.0	0.0	0.1	0.4	0.2	0.2	0.1	0.1
**Window**									
Average	4.5 (± 1.8)	5.0 (± 2.6)	4.8 (± 2.9)	4.5 (± 2.3)	4.9 (± 2.7)	5.3 (± 2.3)	5.4 (± 2.9)	4.4 (± 2.1)	4.8 (± 2.5)
Average screened window	0.2	0.2	0.1	0.3	0.5	0.4	0.4	0.3	0.3
**Eave**									
Eave exist	99.4%	90.5%	85.4%	83.9%	89.4%	84.2%	91.1%	85.2%	89.0%
Screened	19.2%	17.9%	20.7%	31.9%	35.4%	22.4%	36.0%	15.2%	24.9%
**Electricity**									
No electricity	14.3%	0.0%	8.9%	6.5%	2.8%	2.0%	0.6%	33.5%	8.6%
Have electricity	85.7%	100.0%	91.1%	93.5%	97.2%	98.0%	99.4%	66.5%	91.4%
PLN	72.9%	83.2%	73.6%	68.8%	100.0%	61.6%	62.6%	65.0%	74.0%
Solar Cell	17.4%	16.8%	25.0%	3.8%	0.0%	5.1%	16.8%	11.7%	12.6%
Generator	9.7%	0.0%	1.4%	27.4%	0.0%	33.3%	20.7%	23.3%	13.4%

Table S3-3. Suffering from malaria.

Malaria-based data	Keerom n=168	Jayapura n=179	Sarmi n=158	Mimika n=168	Boven Digoel n=142	Yapen Islands n=101	Waropen n=180	Asmat n=155	Total n=1251
**At least one member of the household has been suffered by malaria**	82.7%	78.8%	94.9%	92.3%	82.4%	72.3%	65.0%	69.7%	79.9%
Primary family members	96.4%	96.5%	93.1%	96.8%	84.0%	98.4%	96.3%	96.7%	94.6%
Not primary family members	1.2%	1.4%	4.9%	2.9%	8.4%	1.6%	1.4%	3.3%	3.2%
Answer not filled	2.4%	2.1%	2.0%	0.3%	7.6%	0.0%	2.3%	0.0%	2.1%
**Malaria last incidences**									
< two weeks ago	19.4%	12.8%	6.0%	5.8%	11.1%	12.3%	18.8%	16.7%	12.5%
> two weeks ago AND < one month	27.3%	19.9%	45.3%	27.7%	12.8%	23.3%	37.6%	18.5%	27.3%
> one month	43.9%	65.2%	46.0%	63.9%	70.1%	63.0%	41.9%	57.4%	56.0%
Don't know	9.4%	2.1%	2.7%	2.6%	6.0%	1.4%	1.7%	7.4%	4.2%
**How to know malaria**									
Physician's examination result	23.4%	18.1%	9.3%	22.6%	19.9%	17.2%	18.3%	10.4%	17.5%
Microscopy or RDT result	53.5%	55.6%	53.2%	69.2%	39.8%	45.5%	36.5%	45.2%	49.9%
Symptoms	7.0%	6.2%	22.6%	3.2%	14.6%	14.5%	13.7%	16.3%	12.2%
Previous experience	5.5%	3.7%	8.1%	0.0%	8.9%	8.3%	4.1%	9.0%	5.9%
Notified by someone else	0.4%	0.0%	1.6%	0.0%	3.3%	0.0%	0.0%	0.5%	0.8%
Other	0.8%	0.0%	2.0%	0.0%	4.1%	0.7%	1.7%	4.1%	1.7%
Don't know	9.4%	16.5%	3.2%	5.0%	9.3%	13.8%	25.7%	14.5%	12.1%
**Death caused by malaria in recent two years**	0.6%	0.6%	5.7%	3.0%	2.8%	2.0%	0.6%	1.9%	2.1%

Table S3-4. Mosquito bites prevention efforts.

Mosquito Biting Prevention	Keerom n=168	Jayapura n=179	Sarmi n=158	Mimika n=168	Boven Digoel n=142	Yapen Islands n=101	Waropen n=180	Asmat n=155	Total n=1251
**IRS**									
Have been sprayed	67.3%	34.1%	3.8%	40.5%	22.5%	44.6%	5.0%	12.9%	28.3%
Sprayed < 3 months	32.7%	77.0%	0.0%	61.8%	3.1%	82.2%	0.0%	0.0%	46.3%
Sprayed 3-6 months	6.2%	18.0%	0.0%	25.0%	43.8%	0.0%	11.1%	45.0%	16.7%
Sprayed > 6 months	61.1%	0.0%	66.7%	7.4%	31.3%	13.3%	77.8%	40.0%	30.8%
Sprayed by health workers	100.0%	95.1%	33.3%	94.1%	90.6%	97.8%	88.9%	85.0%	94.6%
Sprayed by NGO	0.0%	0.0%	33.3%	1.5%	0.0%	0.0%	0.0%	0.0%	0.8%
Sprayed by others	0.0%	0.0%	16.7%	0.0%	6.3%	0.0%	0.0%	5.0%	1.1%
**ITNs**									
Bedroom average	2.2 (± 0.9)	2.9 (± 1.3)	2.7 (± 1.3)	2.5 (± 0.9)	2.6 (± 1.4)	2.9 (± 1.2)	2.7 (± 1.3)	2.7 (± 1.4)	2.7 (± 1.2)
Average ITNs coverage	2.2 (± 1.2)	2.7 (± 1.7)	3.0 (± 1.5)	2.5 (± 1.5)	2.3 (± 1.6)	3.1 (± 1.8)	2.5 (± 1.8)	2.8 (± 1.8)	2.6 (± 1.6)
Everybody using net	78.0%	72.1%	79.1%	70.2%	54.2%	67.3%	73.3%	63.2%	70.2%
Slept under net lastnight	3.6 (84.0%)	3.7 (76.3%)	4.5 (78.5%)	4.2 (70.8%)	3.3 (58.8%)	3.7 (59.6%)	3.5 (77.0%)	4.8 (68.5%)	3.9 (72.7%)
ITN Access	84.0%	82.2%	79.3%	72.6%	63.1%	78.5%	80.9%	68.5%	75.7%
**Households with ITNs questions**	**n = 0**	**n = 174**	**n = 155**	**n = 151**	**n = 117**	**n = 98**	**n = 161**	**n = 150**	**n = 1006**
**ITN age**									
> 3 years	-	3 (1.7%)	18 (11.6%)	25 (16.6%)	1 (0.9%)	0 (0.0%)	32 (19.9%)	1 (0.7%)	80 (8.0%)
> 1 year and ≤ 3 years	-	80 (46.0%)	108 (69.7%)	85 (56.3%)	23 (19.7%)	0 (0.0%)	48 (29.8%)	24 (16.0%)	368 (36.6%)
≤ 1 year	-	90 (51.7%)	28 (18.1%)	41 (27.2%)	93 (79.5%)	98 (100.0%)	81 (50.3%)	118 (78.7%)	549 (54.6%)
**ITN condition**									
Lots of holes	-	18 (10.3%)	17 (11.0%)	40 (26.5%)	17 (14.5%)	2 (2.0%)	15 (9.3%)	15 (10.0%)	124 (12.3%)
Few holes	-	27 (15.5%)	48 (31.0%)	58 (38.4%)	29 (24.8%)	3 (3.1%)	48 (29.8%)	33 (22.0%)	246 (24.5%)
Good	-	126 (72.4%)	90 (58.1%)	53 (35.1%)	70 (59.8%)	89 (90.8%)	96 (59.6%)	101 (67.3%)	625 (62.1%)

**Reasons of not using ITNs last night**	**n=58**	**n=58**	**n=38**	**n=71**	**n=89**	**n=67**	**n=63**	**n=78**	**n=522**
Do not have bednet	5.2%	3.4%	5.3%	26.8%	24.7%	3.0%	19.0%	6.4%	12.8%
Bednet is not enough	6.9%	0.0%	23.7%	19.7%	19.1%	6.0%	12.7%	29.5%	15.1%
Bednet condition is not good	0.0%	0.0%	0.0%	4.2%	5.6%	0.0%	1.6%	1.3%	1.9%
Do not like the smell of chemical ingredients	1.7%	0.0%	0.0%	1.4%	4.5%	0.0%	4.8%	2.6%	2.1%
Uncomfortable because of feeling hot inside	27.6%	62.1%	44.7%	23.9%	30.3%	46.3%	41.3%	34.6%	37.7%
No mosquito last night	0.0%	15.5%	10.5%	0.0%	5.6%	11.9%	12.7%	9.0%	7.9%
Feeling itching/rash in the skin	0.0%	0.0%	0.0%	0.0%	0.0%	3.0%	0.0%	1.3%	0.6%
Feeling burned inside the bednet	0.0%	0.0%	0.0%	1.4%	0.0%	0.0%	0.0%	0.0%	0.2%
Not at home last night	10.3%	3.4%	0.0%	14.1%	0.0%	0.0%	0.0%	6.4%	4.4%
Other reasons	8.6%	13.8%	15.8%	2.8%	7.9%	4.5%	4.8%	2.6%	6.9%
Don't know	39.7%	1.7%	0.0%	5.6%	2.2%	25.4%	3.2%	6.4%	10.3%
**Using insect repellent indoor**	107 (63.7%)	82 (45.8%)	56 (35.4%)	72 (42.9%)	96 (67.6%)	34 (33.7%)	104 (57.8%)	77 (49.7%)	628 (50.2%)
Mosquito coil	69.0%	58.4%	61.6%	69.0%	70.7%	46.3%	51.5%	85.4%	64.7%
Mosquito spray	20.6%	36.0%	26.0%	23.8%	21.6%	31.7%	41.2%	12.2%	26.9%
Emanator	4.8%	0.0%	1.4%	0.0%	5.2%	9.8%	1.5%	1.2%	2.7%
Personal repellent	5.6%	4.5%	11.0%	6.0%	0.9%	12.2%	5.1%	1.2%	5.1%

Table S3-5. Human behavior at night.

Human Behavior at Night	Keerom n=168	Jayapura n=179	Sarmi n=158	Mimika n=168	Boven Digoel n=142	Yapen Islands n=101	Waropen n=180	Asmat n=155	Total n=1251
**Dinner time**									
Before 7.00 pm	26.8%	22.9%	11.4%	11.3%	16.2%	29.7%	10.0%	21.9%	18.2%
7.00 pm - 9.00 pm	69.0%	74.3%	85.4%	87.5%	81.0%	62.4%	85.6%	65.8%	77.1%
After 9.00 pm	2.4%	1.7%	2.5%	0.6%	2.8%	6.9%	3.9%	3.2%	2.8%
**Dinner outside house**	3.0%	2.8%	4.4%	3.6%	1.4%	4.0%	2.8%	4.5%	3.3%
**Resting outside after dinner**	16.7%	16.8%	31.0%	32.7%	11.3%	47.5%	11.7%	18.1%	22.0%
**Go outside at night**	44.0%	50.3%	50.0%	35.7%	45.1%	36.6%	44.4%	60.0%	46.1%
Go to neighborhood	59.5%	85.6%	73.4%	95.0%	40.6%	56.8%	83.8%	68.8%	71.8%
Go hunting/fishing	0.0%	12.2%	21.5%	0.0%	48.4%	18.9%	7.5%	6.5%	16.3%
Others	18.9%	2.2%	5.1%	5.0%	10.9%	24.3%	8.8%	24.7%	12.0%
**Go outside > 1 hour**	78.4%	62.2%	74.7%	55.0%	90.6%	62.2%	58.8%	67.7%	68.8%
**Using repellent outside**	2.7%	5.6%	8.9%	3.3%	18.8%	13.5%	28.8%	7.5%	10.9%
**Sleeping time**									
Before 7.00 pm	1.2%	0.6%	0.6%	0.0%	0.7%	0.0%	0.0%	2.6%	0.7%
7.00 pm - 9.00 pm	29.8%	36.9%	30.4%	16.7%	19.0%	26.7%	27.8%	14.8%	25.5%
After 9.00 pm	69.0%	62.0%	69.0%	83.3%	80.3%	72.3%	72.2%	82.6%	73.6%
**Waking up time**									
Before 6.00 am	64.9%	89.9%	65.8%	58.3%	66.2%	50.5%	76.7%	59.4%	67.7%
6.00 am - 7.00 am	28.0%	8.9%	27.2%	39.3%	27.5%	24.8%	19.4%	36.1%	26.1%
After 7.00 am	7.1%	0.6%	7.0%	2.4%	6.3%	24.8%	3.9%	4.5%	6.1%
**Sleep outside house**	0.0%	0.6%	0.0%	0.0%	0.0%	1.0%	1.1%	0.6%	0.4%
**sleep under bednet**	92.3%	82.7%	89.9%	79.2%	66.2%	76.2%	83.9%	78.7%	81.7%
